# Cold Stress‐Induced (*Z*)‐3‐Hexenol and Thymol Enhance Cold Tolerance of Tea Plants by Activating Ca^2+^ Signalling

**DOI:** 10.1111/pbi.70346

**Published:** 2025-09-03

**Authors:** Yuantao Liu, Yaling Song, Zhengwei Luo, Lisha Wang, Jieyang Jin, Tingting Jing, Mingyue Zhao, Qiang Wang, Wilfried Schwab, Meng Ye, Chuankui Song

**Affiliations:** ^1^ National Key Laboratory for Tea Plant Germplasm Innovation and Resource Utilization Anhui Agricultural University Hefei China; ^2^ Tea Research Institute Chinese Academy of Agricultural Sciences Hangzhou China; ^3^ Biotechnology of Natural Products Technische Universität München Freising Germany

**Keywords:** (*Z*)‐3‐hexenol, Ca^2+^ signalling, cold stress, Fluo‐8, tea plants, thymol, VOCs

## Abstract

Volatile organic compounds (VOCs) released under cold stress have emerged as important mediators of stress tolerance. However, the specific functional VOCs and the mechanisms through which they confer cold tolerance remain largely unknown. In this study, we established a Fluo‐8‐based calcium detection system in tea ( *Camellia sinensis* ) protoplasts to investigate the interplay between cold‐induced VOCs, calcium signalling, and cold tolerance. We identified (*Z*)‐3‐hexenol and thymol as key VOCs that significantly enhance tea plant cold tolerance by activating cytosolic calcium signalling. These VOCs upregulated cold‐responsive genes (*CsICE1*, *CsCBF1*, and *CsCBF2*), enhanced antioxidant enzyme activities (SOD and POD), and improved photosynthetic efficiency under cold stress. Furthermore, we revealed that *CsCDPK4*, a calcium‐dependent protein kinase, acts as a key mediator of (*Z*)‐3‐hexenol and thymol‐induced calcium signalling. Silencing *CsCDPK4* abolished the beneficial effects of (*Z*)‐3‐hexenol and thymol, underscoring its critical role in translating calcium signals into physiological adaptations. Our findings provide a mechanistic framework linking VOC perception, calcium signalling, and cold stress tolerance, offering novel strategies for enhancing crop resilience against abiotic stress in the face of climate challenges.

## 1 Introduction

Cold stress is among the major environmental factors that significantly impact plant growth and development. In response, plants activate a suite of defence mechanisms spanning molecular to physiological adaptations to mitigate cold stress (Ding et al. 2019; Kidokoro et al. 2022; Wang et al. 2024). Among these responses, the Inducer of CBF Expression (ICE) and its downstream C‐repeat Binding Factors (CBFs) are rapidly induced by cold stress, forming a regulatory module that plays a central role in cold acclimation across various plant species (Shi et al. 2018; Ding et al. 2019; Zhang et al. 2021; Adhikari et al. 2022; Wu et al. 2022). Additionally, protective antioxidant enzymes, such as superoxide dismutase (SOD) and peroxidase (POD), are elevated to reduce oxidative damage caused by cold exposure (Smirnoff and Arnaud 2019; Mittler et al. 2022).

Beyond these well‐studied responses, emerging studies indicate that plants under cold stress release specific volatile organic compounds (VOCs) that prime enhanced cold tolerance, thereby reducing cold‐induced damage. For example, green leaf volatiles (GLVs) have been shown to protect maize ( *Zea mays* ) seedlings from cold stress (Cofer et al. 2018). In tea plants, cold‐induced nerolidol, geraniol, linalool, and methyl salicylate (MeSA) play crucial roles in priming cold tolerance (Zhao, Wang, et al. 2020). Despite this promising progress, the specific VOCs that mediate plant responses to cold stress remain largely unidentified, and the mechanisms through which these VOCs confer cold tolerance are still poorly understood.

Cold stress is also known to trigger increases in cytosolic Ca^2+^ concentration ([Ca^2+^]_cyt_) (Liu et al. 2021; Wang et al. 2021; Zhang et al. 2021; Wu et al. 2022). Various Ca^2+^ sensors, including calmodulins (CaMs), calmodulin‐like proteins (CMLs), Ca^2+^‐dependent protein kinases (CDPKs), calcineurin B‐like proteins (CBLs), and their interacting kinases (CIPKs), decode these Ca^2+^ signals, forming intricate signalling networks that enable plants to mount specific, robust defence responses (Liu et al. 2018; Luan and Wang 2021; Allan et al. 2022; Ding et al. 2022). Some evidence suggests that Ca^2+^ signalling plays a critical role in VOC‐induced defences. For instance, exposure to the GLV (*Z*)‐3‐hexenyl acetate increases cytosolic Ca^2+^ flux in tomato plants (Zebelo et al. 2012), while (*E*)‐2‐hexenal promotes cytosolic Ca^2+^ transients in Arabidopsis, likely by enhancing reactive oxygen species (ROS) production and activating ROS‐responsive Ca^2+^ channels (Asai et al. 2009). Similarly, indole boosts Ca^2+^ signalling pathways by upregulating the expression of *CML*, *CDPK*, and *CIPK* genes (Ye et al. 2021). Although the role of Ca^2+^ signalling in VOC‐induced defences is increasingly understood, whether Ca^2+^ signalling also contributes to VOC‐mediated cold tolerance remains unknown.

Using transgenic Arabidopsis expressing the GCaMP3 fluorescent protein‐based Ca^2+^ sensor (Toyota et al. 2018), Aratani et al. identified two GLVs, (*Z*)‐3‐hexenal and (*E*)‐2‐hexenal, that increase [Ca^2+^]_cyt_ in Arabidopsis. They further demonstrated that these volatiles trigger the expression of biotic and abiotic stress‐responsive genes (Aratani et al. 2023). This approach provides a powerful tool for screening and identifying Ca^2+^‐dependent active volatiles, and for investigating the molecular basis of VOC‐mediated signalling mechanisms. However, this advanced system relies heavily on stable transformation for fluorescence reporter generation and on real‐time Ca^2+^ imaging, making it challenging to apply in plants that lack stable transformation technology. Furthermore, achieving clear fluorescence imaging is difficult in leaves with thick wax layers, such as in tea plants. Therefore, there is a pressing need for a more practical method that can be broadly applied to non‐transformable plants.

In this study, using tea plants as a model, we first examined the connections between cold‐induced VOCs, calcium signalling, and cold tolerance. We then developed a real‐time detection system of cellular calcium dynamics in tea cells using a Fluo‐8 dye (Fluo‐8/AM, the acetoxymethyl ester form of Fluo‐8). With this high‐throughput system, we identified active volatiles, including (*E*)‐2‐hexenal, (*Z*)‐3‐hexenol, thymol, and α‐farnesene. Finally, through pharmacological manipulation and reverse genetic approaches, we elucidated the mechanism by which (*Z*)‐3‐hexenol and thymol enhance cold tolerance by activating Ca^2+^ signalling. Our findings uncover specific VOCs and Ca^2+^‐dependent signalling pathways that can be targeted to improve cold resilience in crops, and offer a feasible method for VOC screening in plants that lack stable transformation capabilities. This research offers a novel framework for understanding the mechanistic role of VOCs in mediating cold tolerance in plants, representing a significant step forward in the development of stress‐resilient agricultural systems in the face of climate challenges.

## 2 Results

### 2.1 Calcium Signalling Is Essential for VOC‐Induced Cold Tolerance in Tea Plants

**FIGURE 1:**
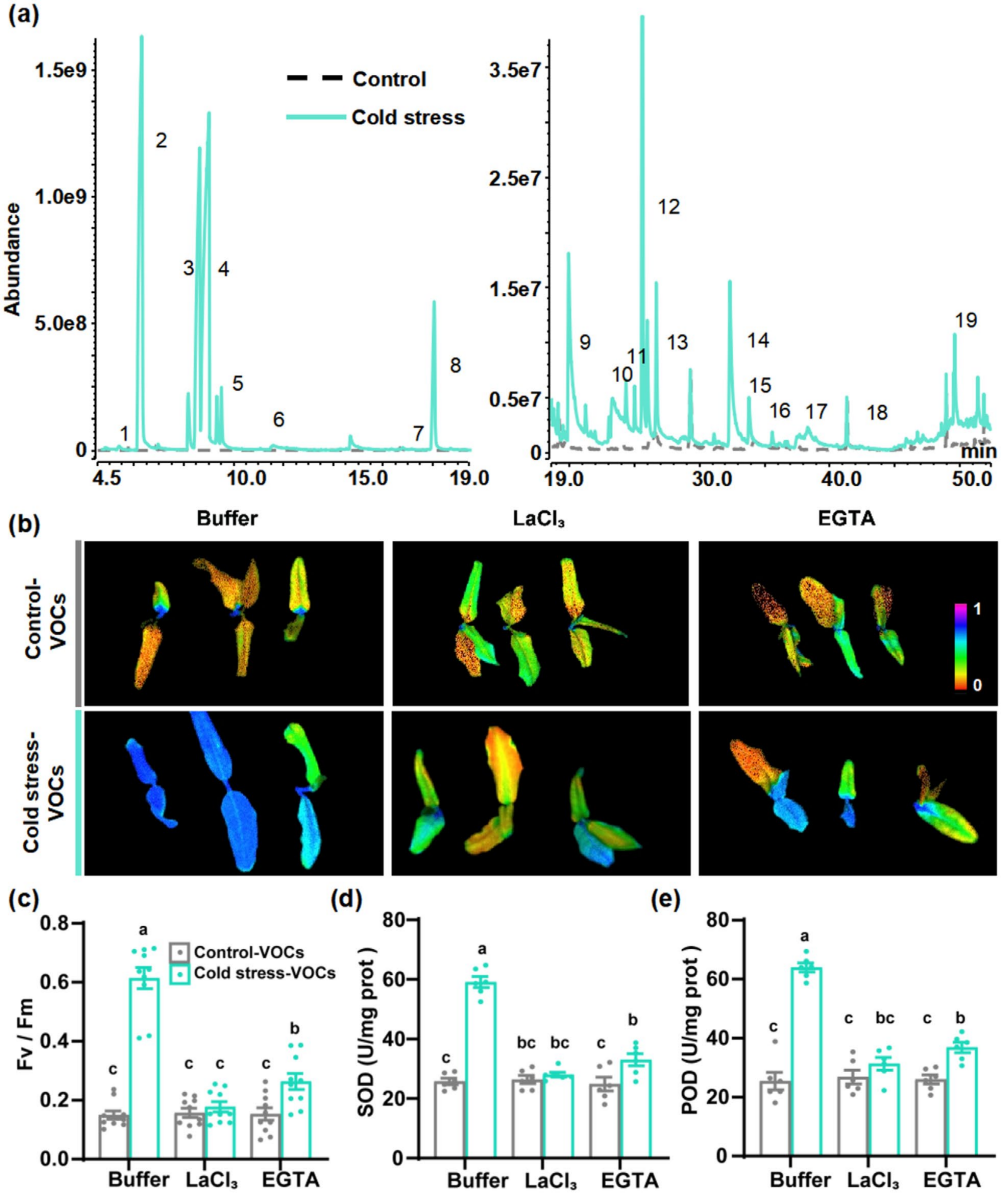
Calcium signalling is likely involved in mediating VOC‐induced cold tolerance in tea plants. (a) GC/MS total ion chromatograms of VOCs released during the recovery phase of tea plants following cold stress. For detailed VOC information, see Table S1. (b) Chlorophyll fluorescence images of tea plants pre‐treated with calcium inhibitors LaCl_3_ or EGTA, exposed to VOCs emitted from either control or cold‐stressed plants, and subsequently subjected to cold stress. Fv/Fm is presented using a pseudo‐colour scale ranging from 0 to 1. Higher Fv/Fm values (purple blue colours) indicate less damage to photosystem II caused by cold stress. The experimental design is outlined in Figure S1. (c–e) Measurements of Fv/Fm (c), SOD activity (d), and POD activity (e) in tea plants pre‐treated with calcium inhibitors LaCl_3_ or EGTA, exposed to VOCs emitted from either control or cold‐stressed plants, and subsequently subjected to cold stress (±SE; *n* = 10 plants for c, *n* = 6 plants for d and e). Data points represent individual replicates. Different letters indicate significant differences at *p* < 0.05 (two‐way ANOVA with Tukey's HSD post hoc test).

To investigate the relationships between VOCs, calcium signalling, and cold tolerance, we first profiled VOCs released by tea plants during the recovery phase following cold treatment. This analysis identified 19 VOCs that significantly increased post‐cold treatment (Figure 1a; Table S1). Among these, geraniol, linalool, and MeSA are known to enhance cold stress resistance in tea plants (Zhao, Wang, et al. 2020). This led us to hypothesise that these cold‐induced VOCs may play a regulatory role in tea plant cold tolerance. To test this, healthy tea plants were exposed to the full VOC blend released from cold‐stressed plants, and subsequently subjected to cold stress (Figure S1). Compared to plants exposed to VOCs from unstressed controls, those treated with cold‐induced VOCs exhibited markedly improved cold tolerance, as evidenced by higher maximum quantum efficiency of photosystem II photochemistry (Fv/Fm) (Figure 1b,c). Fv/Fm reflects the functional status of photosystem II; higher values indicate less damage caused by cold stress and thus stronger physiological resilience. Additionally, the plants exposed to cold‐induced VOCs gained increased antioxidant enzyme activities of SOD and POD (Figure 1d,e).

Calcium signalling has been previously shown to respond to VOCs (Asai et al. 2009; Zebelo et al. 2012; Ye et al. 2021; Aratani et al. 2023). To determine whether calcium signalling is involved in VOC‐induced cold tolerance in tea plants, we pre‐treated plants with LaCl_3_ and EGTA, inhibitors that block calcium channels and chelate calcium, respectively. To exclude potential negative effects of these inhibitors on tea plants, we first treated plants with LaCl_3_ or EGTA alone and then evaluated their impact on VOC‐induced cold responses under both cold stress and ambient conditions. The results showed that treatment with these inhibitors alone had no significant effect on Fv/Fm or on the activities of SOD and POD (Figure S2), suggesting that the inhibitors themselves did not adversely affect key physiological parameters in tea plants. However, pre‐treatment with LaCl_3_ or EGTA substantially reduced VOC‐induced cold tolerance, as evidenced by significant decreases in Fv/Fm and the activities of SOD and POD following cold exposure (Figure 1b–e). Furthermore, under non‐stress conditions, the VOC‐induced increases in SOD and POD activities were also significantly suppressed by calcium inhibition, whereas Fv/Fm remained unchanged (Figure S3). These results indicate that calcium signalling is likely involved in mediating VOC‐induced cold tolerance in tea plants.

### 2.2 Real‐Time Detection of Cellular Calcium Dynamics in Tea Leaf Cells Using Fluo‐8

**FIGURE 2:**
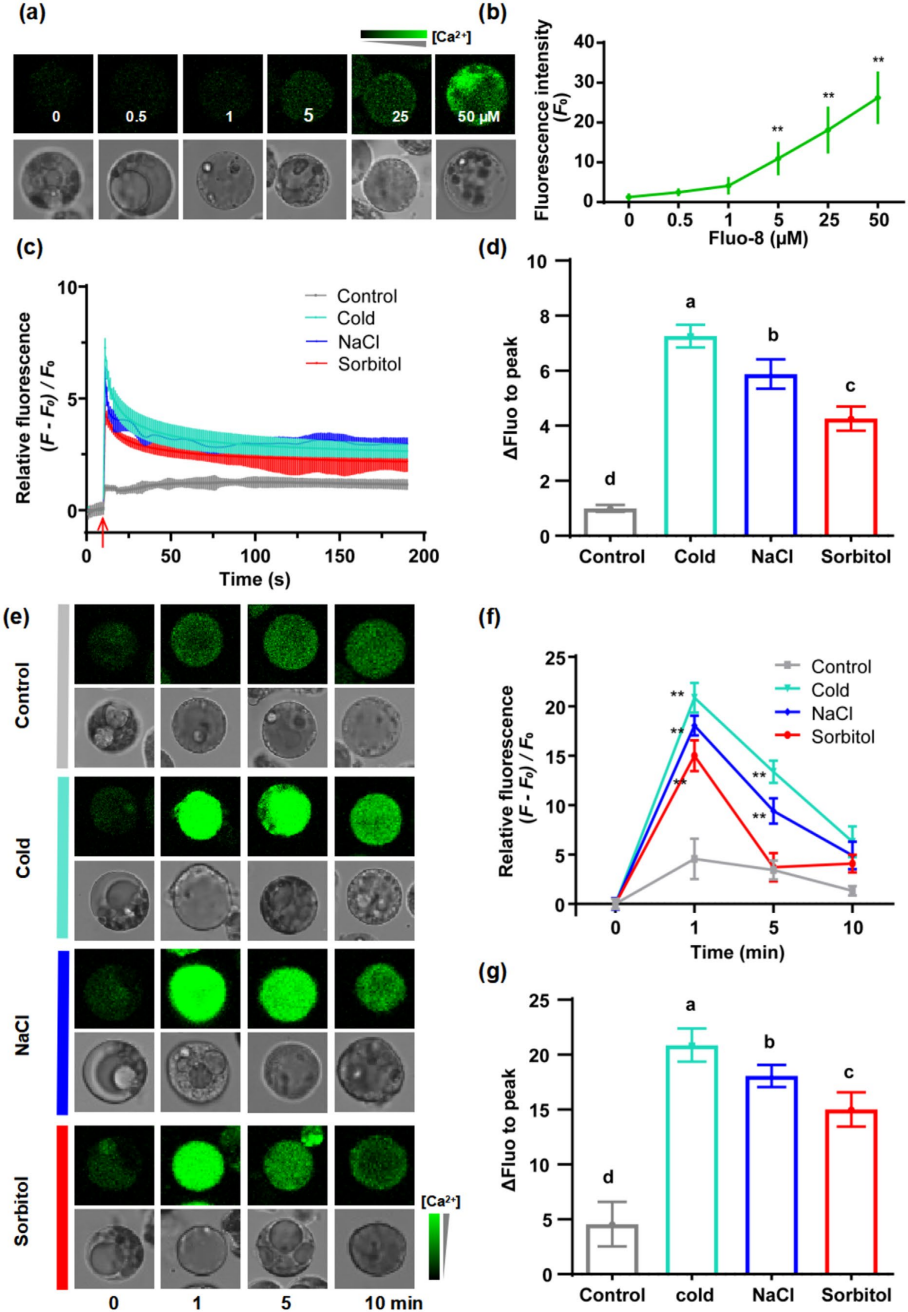
Real‐time detection of cellular Ca^2+^ dynamics in tea leaf cells using Fluo‐8 dye under different stress conditions. (a) Confocal imaging of background [Ca^2+^]_cyt_ fluorescence (*F*_0_) in tea protoplast loaded with different concentrations of Fluo‐8. Green fluorescence signals are presented using a pseudo‐colour scale to indicate the cytosolic Ca^2+^ levels. (b) Quantification of fluorescence intensity in protoplasts treated with different Fluo‐8 concentrations presented in (a) (±SD; *n* = 30 protoplast cells). Asterisks indicate significant differences compared to the control (0 μM Fluo‐8) (**p < 0.01, Student's *t*‐test). (c) Time‐course quantification of [Ca^2+^]_cyt_ dynamics in tea protoplasts subjected to cold (0°C), salt (0.5 M NaCl), and hyper‐osmotic stress (1 M sorbitol). Luminescence was recorded at 1‐s intervals using a multimode microplate reader (±SE; *n* = 8 replicates). The red arrow indicates the time point of reagent addition and reaction initiation. The relative fluorescence intensity, calculated as (*F–F*_0_)/*F*_0_, was determined by normalising the fluorescence intensity at each time point (*F*) to the baseline fluorescence intensity (*F*_0_). (d) Comparison of the maximal Ca^2+^ fluorescence intensity under each stress treatment shown in (c). Different letters indicate statistically significant differences at *p* < 0.05 (two‐way ANOVA with Tukey's H‐SD post hoc test). (e) Confocal imaging of [Ca^2+^]_cyt_ in protoplasts under cold (0°C), salt (0.5 M NaCl), and hyper‐osmotic stress (1 M sorbitol). Fluorescence is scaled by pseudo‐colour. (f) Quantification of relative Ca^2+^ fluorescence intensity from (e). Asterisks indicate significant differences between the treatment and control at each time point (**p < 0.01, Student's *t*‐test). (g) Comparison of the maximal Ca^2+^ fluorescence intensity changes under different stress conditions shown in (e). Different letters indicate significant differences at *p* < 0.05 (two‐way ANOVA with Tukey's HSD post hoc test).

To assess the responsiveness of calcium signalling to VOCs, we developed a method to detect real‐time changes in cellular calcium levels. Given limitations in stable transgenic technology for tea plants, direct observation of calcium‐sensitive fluorescence signals from transgenic lines is not feasible. Instead, we utilised tea leaf protoplasts as a detection system (Figure S4), applying the calcium‐sensitive fluorescent dye Fluo‐8 to monitor Ca^2+^ dynamics. We first optimised Fluo‐8 concentrations for signal detection. Fluorescence intensity was comparable to control levels at 0.5 and 1 μM, but increased markedly at 5 μM, with even stronger signals observed at 25 and 50 μM (Figure 2a,b). To balance sensitivity and minimise background fluorescence, we selected 5 μM Fluo‐8 for subsequent experiments.

To validate this Ca^2+^ detection system, we combined Fluo‐8 with a fully automated fluorescence microplate reader capable of high‐frequency acquisition (1‐s intervals). Tea protoplasts were exposed to three abiotic stressors: cold (0°C), salt (0.5 M NaCl), and hyper‐osmotic stress (1 M sorbitol), all of which are known to rapidly elevate cytosolic Ca^2+^ levels (Yuan et al. 2014; Ma et al. 2015; Jiang et al. 2019). Each treatment triggered a rapid rise in [Ca^2+^]_cyt_ to a peak within seconds, followed by a gradual return to baseline levels (Figure 2c), consistent with previous reports (Yuan et al. 2014; Ma et al. 2015; Jiang et al. 2019). These stress‐induced Ca^2+^ transients were significantly attenuated by pre‐treatment with calcium inhibitors LaCl_3_ or EGTA (Figure S5), confirming the Ca^2+^‐specific nature of the observed signals. Notably, the fluorescence intensity change was significantly higher in NaCl‐treated cells than in sorbitol‐treated cells at equivalent osmolality, indicating that NaCl's ionic effect may further elevate [Ca^2+^]_cyt_ (Figure 2c,d) (Jiang et al. 2019; Pei et al. 2022). This difference was readily and reproducibly detected by our Fluo‐8‐based system, highlighting its sensitivity and reliability in distinguishing nuanced Ca^2+^ responses under varying stress conditions.

To complement the real‐time, high‐frequency acquisition of Ca^2+^ dynamics obtained via fluorescence microplate assays, we also employed confocal laser scanning microscopy to visualise calcium fluorescence intensity under the aforementioned stress treatments at defined time points, providing intuitive and supportive confirmation of the observed Ca^2+^ dynamics (Figure 2e–g). Collectively, these results demonstrate that our Fluo‐8‐based detection system enables accurate, real‐time monitoring of [Ca^2+^]_cyt_ dynamics in tea protoplasts in response to diverse stresses, laying the foundation for subsequent analyses of VOC‐triggered calcium signalling.

### 2.3 Screening of Cold Stress‐Induced Volatiles Using a Fluo‐8 Detection System

**FIGURE 3:**
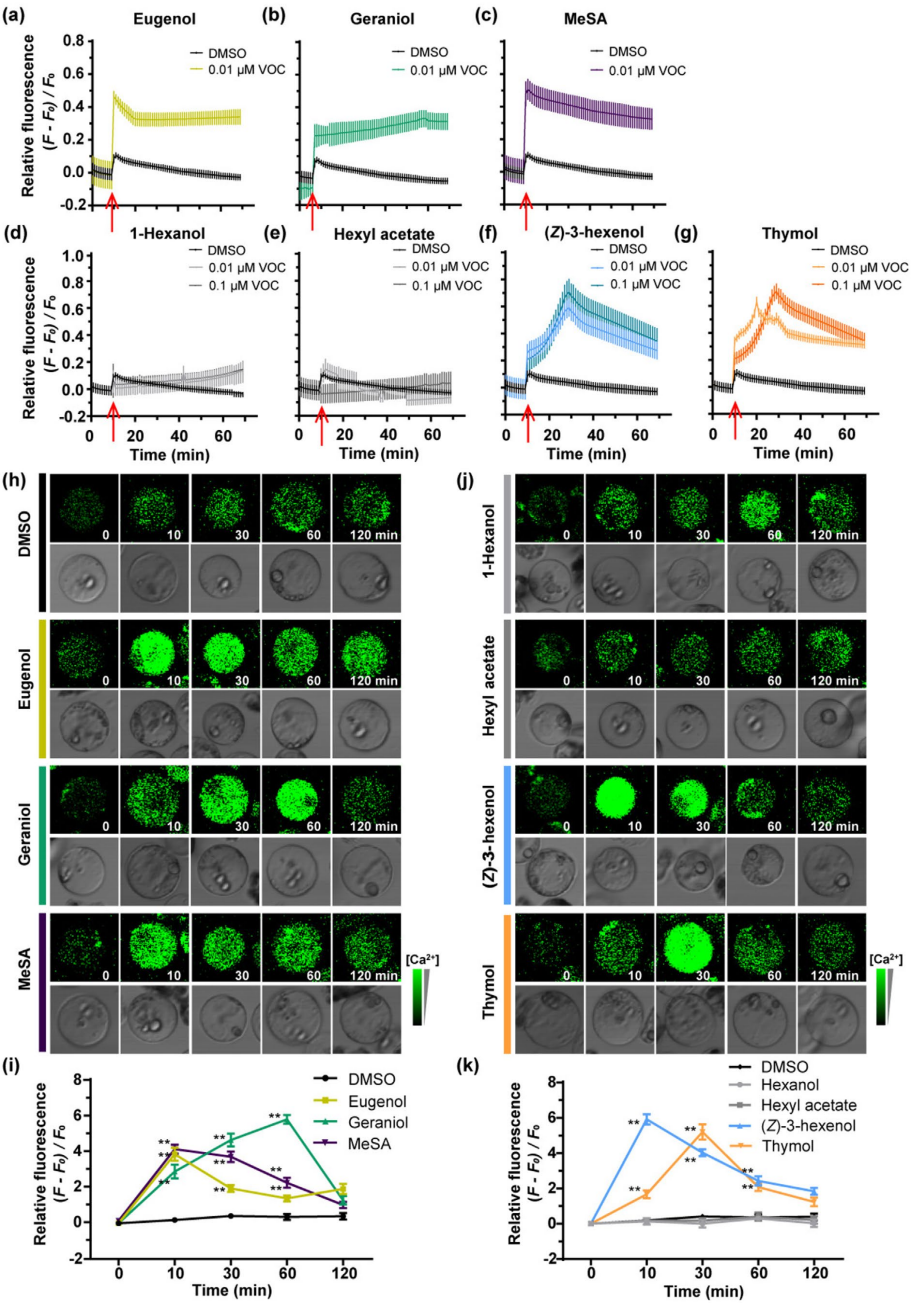
Effects of cold stress‐induced volatiles on Ca^2+^ dynamics in tea leaf protoplasts. (a–g) Time‐course quantification of [Ca^2+^]_cyt_ dynamics in tea protoplasts treated with individual cold‐induced volatiles: Eugenol (a), geraniol (b), MeSA (c), 1‐hexanol (d), hexyl acetate (e), (*Z*)‐3‐hexenol (f) and thymol (g). Fluo‐8 dye was used for detection, and DMSO served as the negative control. Luminescence was recorded at 1‐min intervals using a multimode microplate reader (±SE, *n* = 4 replicates). The relative fluorescence intensity, calculated as (*F–F*_0_)/*F*_0_, was determined by normalising the fluorescence intensity at each time point (*F*) to the baseline fluorescence intensity (*F*_0_). The red arrow indicates the time point of reagent addition and reaction initiation. (h) Confocal imaging of [Ca^2+^]_cyt_ in protoplasts treated with 0.01 μM of geraniol, eugenol, or MeSA. [Ca^2+^]_cyt_ is displayed using a pseudo‐colour scale. (i) Quantification of Ca^2+^ relative fluorescence intensity from (h) (±SE; *n* = 30 protoplast cells). (j) Confocal imaging of [Ca^2+^]_cyt_ in protoplasts treated with 0.01 μM of 1‐hexanol, hexyl acetate, (*Z*)‐3‐hexenol, or thymol. (k) Quantification of Ca^2+^ relative fluorescence intensity from (j) (±SE; *n* = 30 protoplast cells). Relative fluorescence intensity was calculated as described above. Asterisks indicate significant differences between the treatment and control at the corresponding time points (**p < 0.01, Student's *t*‐test).

To assess the Fluo‐8 detection system's effectiveness for capturing VOC‐induced calcium responses, we monitored the [Ca^2+^]_cyt_ changes in tea leaf protoplast cells in response to three known VOCs: eugenol, geraniol, and MeSA, each previously reported to enhance cold tolerance in tea plants (Zhao, Wang, et al. 2020). Luminescence was recorded at 1‐min intervals using a multimode microplate reader. All three VOCs exhibited similar calcium response profiles, characterised by a gradual increase in [Ca^2+^]_cyt_ that peaked and subsequently declined (Figure 3a–c). Additionally, we also performed confocal laser scanning microscopy at 0, 10, 30, 60, and 120 min post‐treatment. The fluorescence imaging confirmed a comparable trend (Figure 3h,i). Notably, the timing of the peak responses varied among the VOCs: eugenol and MeSA induced a rapid calcium peak shortly after treatment, whereas geraniol induced a delayed peak at approximately 60 min. These results indicate that individual VOCs trigger distinct calcium signatures, demonstrating the system's sensitivity and reliability for identifying candidate functional volatiles.

Following this validation, we screened ten of the 19 cold‐induced VOCs from tea plants using the Fluo‐8 system: (*E*)‐2‐hexenal, (*Z*)‐3‐hexenol, 1‐hexanol, (*E*)‐3‐hexenyl acetate, hexyl acetate, 1,2,6‐hexanetriol, nonanal, (*Z*)‐3‐hexenyl 3‐methylbutanoate, thymol, and α‐farnesene. Among these, four VOCs, (*Z*)‐3‐hexenol, thymol, (*E*)‐2‐hexenal, and α‐farnesene, elicited significant increases in [Ca^2+^]_cyt_ (Figures 3d–g and S6). Based on their chemical classification, (*Z*)‐3‐hexenol and (*E*)‐2‐hexenal are GLVs, while thymol and α‐farnesene are terpenoids. To ensure representative coverage of both compound classes and comparable calcium signalling activity, we selected (*Z*)‐3‐hexenol and thymol for further calcium imaging analysis. Additionally, 1‐hexanol and hexyl acetate were included as potential negative controls. Protoplasts treated with (*Z*)‐3‐hexenol and thymol showed gradual increases in Ca^2+^ fluorescence, peaking before returning to baseline, with (*Z*)‐3‐hexenol peaking at approximately 10 min while thymol at around 30 min. As expected, 1‐hexanol and hexyl acetate did not significantly affect Ca^2+^ fluorescence (Figure 3j,k). Additionally, pre‐treatment with the calcium inhibitors LaCl_3_ or EGTA significantly suppressed calcium responses to (*Z*)‐3‐hexenol and thymol (Figure S7), further confirming that the observed fluorescence changes reflect VOC‐induced calcium signalling. These findings indicate that specific cold‐induced VOCs can trigger distinct [Ca^2+^]_cyt_ fluctuations in tea protoplasts, supporting their potential role in enhancing cold tolerance.

### 2.4 [Ca^2+^]_cyt_‐Responsive VOCs Enhance Cold Tolerance in Tea Plants

**FIGURE 4:**
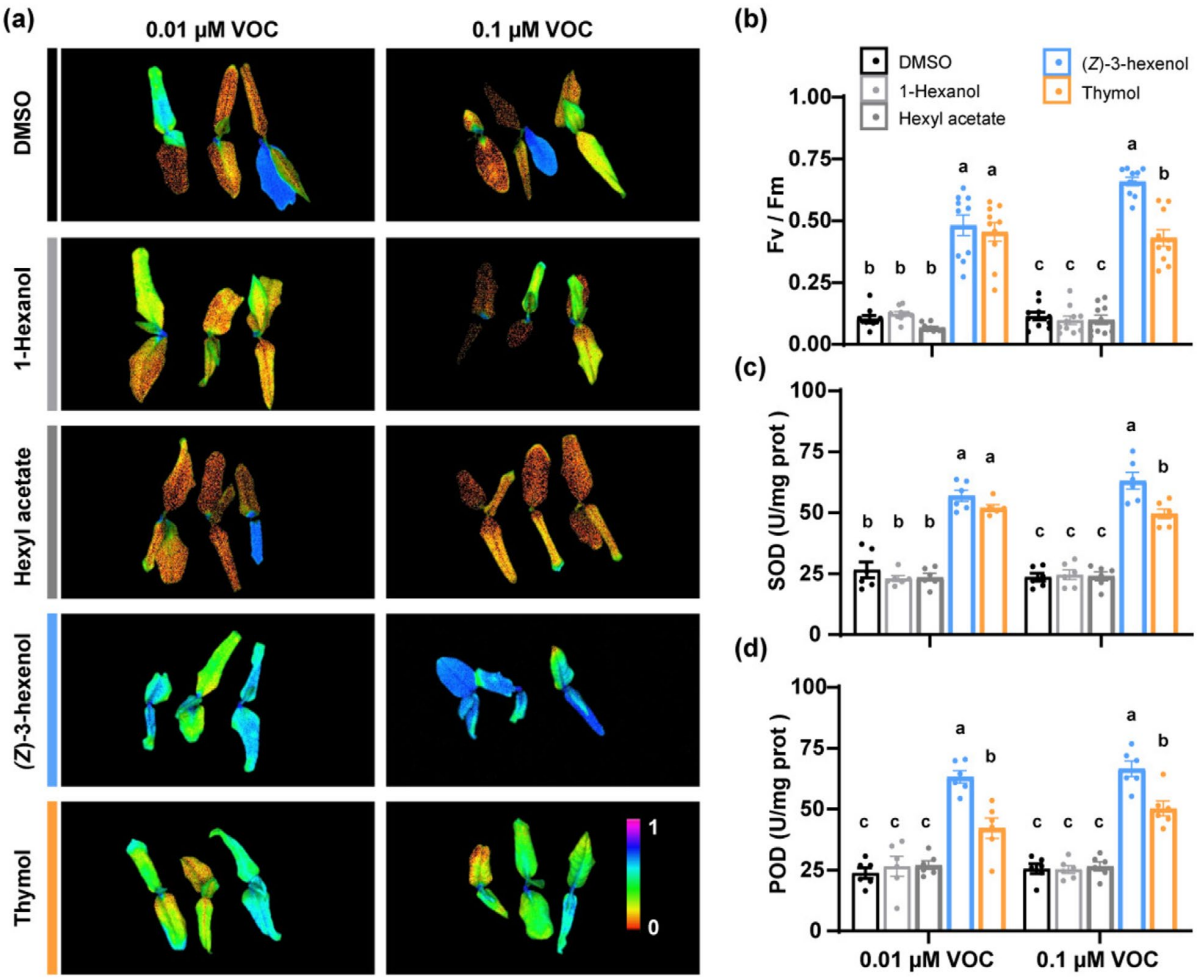
[Ca^2+^]_cyt_‐responsive volatiles enhance cold tolerance in tea plants. (a) Chlorophyll fluorescence images of tea plants exposed to 1‐hexanol, hexyl acetate, (*Z*)‐3‐hexenol, or thymol and subsequently subjected to cold stress. DMSO was used as a negative control. Fv/Fm is presented using a pseudo‐colour scale ranging from 0 to 1. Higher Fv/Fm values (purple blue colours) indicate less damage to photosystem II caused by cold stress. (b–d) Measurements of Fv/Fm (b), SOD activity (c), and POD activity (d) in tea plants treated with DMSO, 1‐hexanol, hexyl acetate, (*Z*)‐3‐hexenol, or thymol and subsequently subjected to cold stress (±SE; *n* = 10 plants for Fv/Fm, *n* = 6 plants for SOD and POD activity). Data points represent individual replicates. Different letters indicate significant differences at *p* < 0.05 (two‐way ANOVA with Tukey's HSD post hoc test).

As demonstrated above, [Ca^2+^]_cyt_ is responsive to (*Z*)‐3‐hexenol and thymol but not to 1‐hexanol or hexyl acetate. To assess the ability of these volatiles to improve cold stress tolerance, we exposed tea plants to these compounds at concentrations matching those naturally released by tea plants under cold stress (Figures 1a and S8). After exposure, the plants were subjected to −5°C in controlled incubators. Plants treated with (*Z*)‐3‐hexenol and thymol showed significantly higher Fv/Fm values compared to those treated with 1‐hexanol, hexyl acetate, or the control group, indicating less damage of photosynthetic system under cold stress (Figure 4a,b). Additionally, antioxidant enzyme activities (SOD and POD) were significantly elevated in the (*Z*)‐3‐hexenol and thymol groups, reflecting enhanced oxidative stress management (Figure 4c,d).

To determine whether these physiological changes were specific to cold conditions, measured the same parameters in plants exposed to volatiles without subsequent cold treatment. While Fv/Fm remained unaffected, SOD and POD activities were significantly elevated in the (*Z*)‐3‐hexenol and thymol groups but not in the 1‐hexanol or hexyl acetate treatments (Figure S9). This indicates that (*Z*)‐3‐hexenol and thymol may prime the antioxidant system even in the absence of cold stress. Together, these results demonstrate that the [Ca^2+^]_cyt_‐responsive volatiles (*Z*)‐3‐hexenol and thymol can effectively increase cold tolerance in tea plants, potentially through improved ROS‐scavenging capacity and preservation of photosynthetic function.

### 2.5 Calcium Signalling Is Required for (*Z*)‐3‐Hexenol and Thymol‐Enhanced Cold Tolerance

**FIGURE 5:**
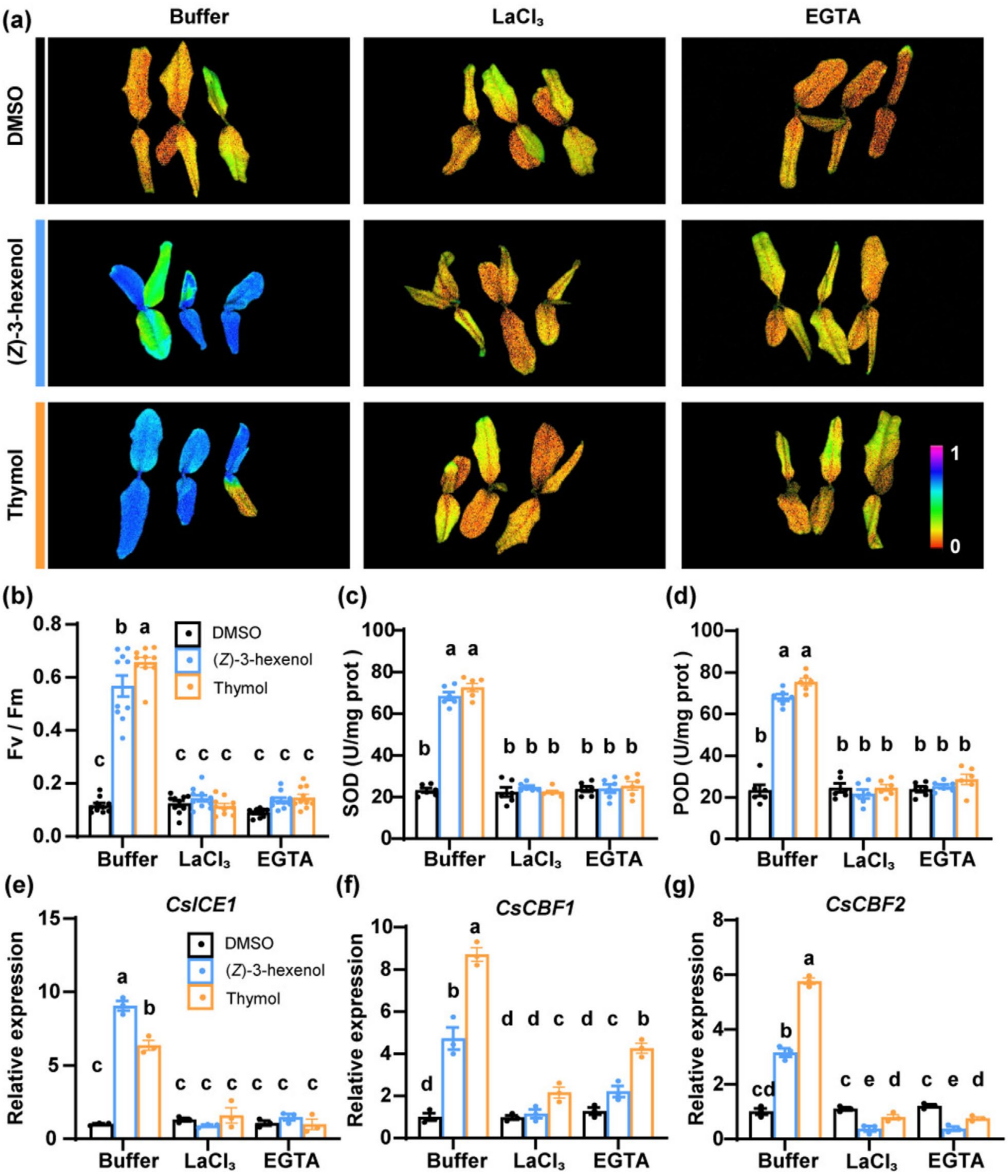
Calcium is crucial for (*Z*)‐3‐hexenol and thymol‐induced cold tolerance in tea plants. (a) Chlorophyll fluorescence images of tea plants pre‐treated with LaCl_3_ or EGTA, then exposed to DMSO, 0.01 μM (*Z*)‐3‐hexenol, or thymol, followed by cold stress. Fv/Fm is presented using a pseudo‐colour scale ranging from 0 to 1. Higher Fv/Fm values (purple blue colours) indicate less damage to photosystem II caused by cold stress. (b–d) Measurements of Fv/Fm (b), SOD activity (c), and POD activity (d) in tea plants pre‐treated with LaCl_3_ or EGTA, then exposed to DMSO, 0.01 μM (*Z*)‐3‐hexenol, or thymol, and subjected to cold stress (± SE; *n* = 10 plants for Fv/Fm, *n* = 6 plants for SOD and POD activity). (e–g) Relative expression levels of cold‐responsive genes *CsICE1* (e), *CsCBF1* (f), and *CsCBF2* (g) in tea plants pre‐treated with LaCl_3_ or EGTA, then exposed to DMSO, 0.01 μM (*Z*)‐3‐hexenol, or thymol, and subjected to cold stress (±SE; *n* = 3 plants). Data points represent individual replicates. Different letters indicate significant differences at *p* < 0.05 (two‐way ANOVA with Tukey's HSD post hoc test).

To investigate the role of calcium signalling in (*Z*)‐3‐hexenol and thymol‐enhanced cold tolerance, we pre‐treated tea plants with the calcium inhibitors LaCl_3_ or EGTA before exposing them to (*Z*)‐3‐hexenol or thymol, and then subjected to cold stress. Plants pre‐treated with these inhibitors exhibited significantly lower Fv/Fm values compared to untreated plants, indicating reduced cold tolerance (Figure 5a,b). Similarly, antioxidant enzyme activities (SOD and POD) were significantly reduced in the LaCl_3_ and EGTA pre‐treated groups (Figure 5c,d). In addition, the expression of cold‐responsive genes *CsICE1*, *CsCBF1*, and Cs*CBF2*, which was significantly upregulated following exposure to (*Z*)‐3‐hexenol and thymol, was markedly reduced in the presence of LaCl_3_ and EGTA after cold stress treatment (Figure 5e–g), supporting the involvement of calcium signalling in volatile‐induced gene expression. These findings demonstrate the critical role of calcium signalling in converting cold‐induced VOC signals into enhanced cold tolerance in tea plants.

### 2.6 CsCDPK4 Translates (*Z*)‐3‐Hexenol‐ and Thymol‐Induced Calcium Signals Into Cold Tolerance

**FIGURE 6:**
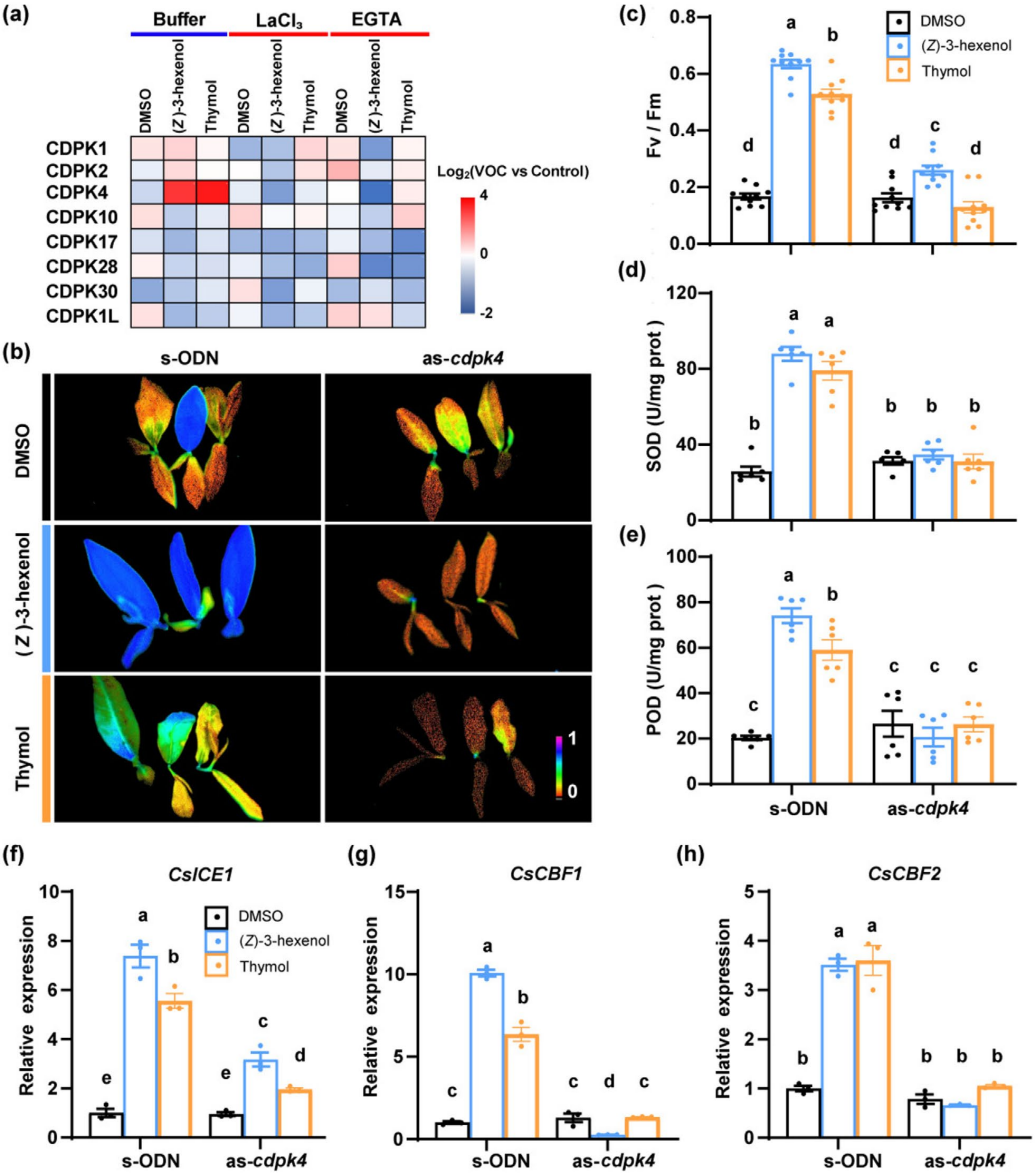
CsCDPK4 is essential for mediating (*Z*)‐3‐hexenol and thymol‐induced cold tolerance in tea plants. (a) Heatmap of *CDPK*s' gene expression in tea plants exposed to (*Z*)‐3‐hexenol or thymol after pre‐treatment with LaCl_3_ or EGTA. The colour scale represents gene expression as log_2_(VOC vs. Control). Data are shown as the mean of three independent experiments. For the full dataset, see Table S2. (b) Chlorophyll fluorescence images of control (s‐ODN) and *CsCDPK4*‐silenced (as*‐cdpk4*) tea plants exposed to (*Z*)‐3‐hexenol or thymol and subsequently subjected to cold stress. Fv/Fm is scaled by pseudo‐colour. (c–e) Measurements of Fv/Fm (c), SOD activity (d), and POD activity (e) in tea plants exposed to (*Z*)‐3‐hexenol or thymol and subsequently subjected to cold stress (±SE; *n* = 10 plants for Fv/Fm, *n* = 6 plants for SOD and POD activity). (f–h) Relative expression levels of cold‐responsive genes *CsICE1* (f), *CsCBF1* (g), and *CsCBF2* (h) in control (s‐ODN) and *CsCDPK4*‐silenced (as*‐cdpk4*) tea plants exposed to (*Z*)‐3‐hexenol or thymol and subsequently subjected to cold stress (±SE; *n* = 3 plants). Data points represent individual replicates. Different letters indicate significant differences at *p* < 0.05 (two‐way ANOVA with Tukey's HSD post hoc test).

Among the major classes of plant Ca^2+^ sensors, including CaMs, CMLs, CBLs/CIPKs, and CDPKs, only CDPKs function both as direct Ca^2+^ sensors and as signal transduction effectors, enabling the rapid conversion of Ca^2+^ signatures into appropriate downstream responses (Yip Delormel and Boudsocq 2019; Ding et al. 2022; Yang et al. 2022). To further elucidate the role of calcium signalling in VOC‐induced cold tolerance, we selected eight significantly upregulated calcium signalling genes from a pool of fourteen cold‐responsive *CDPK* candidates identified through transcriptome analysis (Table S2). We then examined their expression following VOC exposure. Among these eight cold‐inducible *CsCDPK* genes, only *CsCDPK4* showed significantly increased expression following exposure to (*Z*)‐3‐hexenol or thymol. Notably, this effect was attenuated when plants were pre‐treatment with the calcium inhibitors LaCl_3_ and EGTA (Figure 6a).

Using antisense oligonucleotide (as‐ODN) primers, we effectively silenced *CsCDPK4* in tea plants without affecting the expression of other homologous genes (Figure S10). Silencing *CsCDPK4* abolished the cold tolerance effects induced by (*Z*)‐3‐hexenol or thymol, as shown by reduced Fv/Fm values as well as SOD and POD activities (Figure 6b–e). Additionally, the expression of cold‐responsive genes, including *CsICE1*, *CsCBF1*, and *CsCBF2*, was significantly lower in *CsCDPK4*‐silenced plants compared to controls, both under control and cold stress conditions (Figures 6f–h and S11). These results underscore *CsCDPK4*'s role as a critical decoder of Ca^2+^ signals initiated by cold‐induced volatiles, effectively translating these signals into cold resistance responses. This highlights the importance of calcium signalling in mediating VOC perception and activating cold stress defence pathways in tea plants.

## 3 Discussion

Recent evidence suggests that cold‐induced VOCs enhance plant cold tolerance, yet the specific functional VOCs and their underlying mechanisms remain poorly understood (Zhao, Wang, et al. 2020; Jin, Zhao, Jing, Wang, et al. 2023). In this study, we addressed these key knowledge gaps by developing a real‐time calcium detection system in tea protoplasts and systematically investigating the interplay between cold‐induced VOCs, calcium signalling, and cold resistance. Below, we discuss the significance and implications of our findings.

First, our study bridges critical gaps in understanding the functional roles of cold‐induced VOCs and the mechanisms by which they confer cold tolerance. Consistent with previous reports (Zhao, Wang, et al. 2020), we observed that cold stress significantly increases the release of VOCs such as nerolidol, geraniol, linalool, and MeSA, which enhance cold tolerance in tea plants. However, earlier studies did not elucidate how these VOCs confer cold resistance at the molecular level. Although emerging evidence links VOC‐induced resistance with calcium signalling (Ye et al. 2021; Upadhyay et al. 2025), the causal relationship among cold‐induced VOCs, Ca^2+^ signalling, and cold resistance has remained unclear.

Through pharmacological inhibition, we demonstrated that calcium signalling is indispensable for the cold resistance conferred by cold stress‐induced VOCs. Using a combination of high‐throughput calcium detection and genetic approaches, we identified (*Z*)‐3‐hexenol and thymol as key functional VOCs that activate calcium signalling and enhance cold stress adaptation. These VOCs increase the expression of cold‐responsive genes and boost antioxidant enzyme activities, including SOD and POD through activation of calcium signalling, ultimately resulting in enhanced cold tolerance.

The functional roles of (*Z*)‐3‐hexenol and thymol extend beyond cold tolerance. (*Z*)‐3‐hexenol has previously been shown to enhance drought tolerance in tea plants and herbivore resistance in various plant species (Engelberth et al. 2004; Wang et al. 2015; Jing et al. 2019; Jin, Zhao, Jing, Wang, et al. 2023; Sugimoto et al. 2023). Thymol is known to mitigate cadmium stress in tobacco seedlings and confer salt stress tolerance in rice (Ye et al. 2016; Cheng et al. 2020). Our findings expand the functional repertoire of these VOCs, demonstrating their role in enhancing resilience to cold stress.

Second, we identified *CsCDPK4* as a key mediator in this process, underscoring the pivotal role of CDPKs in decoding (*Z*)‐3‐hexenol and thymol‐induced calcium signals. CDPK4 homologues in other plant species are well‐documented mediators of abiotic and biotic stresses. For instance, CDPK29 has been shown to mediate thermotolerance and immunity to *Ralstonia solanacearum* in pepper (Yang et al. 2022), while CPK28 positively regulates plant responses to cold stress in Arabidopsis (Ding et al. 2022). In our study, *CsCDPK4* expression was significantly induced by exposure to (*Z*)‐3‐hexenol or thymol. Silencing *CsCDPK4* abolished the cold tolerance effects of these VOCs, reduced antioxidant enzyme activities, and suppressed the expression of cold‐responsive genes, including *CsICE1*, *CsCBF1*, and *CsCBF2*. However, the observation that (*Z*)‐3‐hexenol‐ and thymol‐induced *CsICE1* expression was not completely abolished in *CsCDPK4*‐silenced plants suggests that other calcium signalling components may also contribute to this process (Jing et al. 2021; Ye et al. 2021; Jiao et al. 2022; Jin, Zhao, Jing, Zhang, et al. 2023). Future studies involving transcriptomic profiling of tea plants treated with (*Z*)‐3‐hexenol or thymol under cold stress may help uncover the downstream regulatory cascades triggered by volatile perception. A more complete understanding of the crosstalk between VOC signalling and calcium‐mediated pathways would significantly advance our knowledge of cold stress responses. Nevertheless, our findings firmly establish CsCDPK4 as a critical node in the signalling cascade, translating (*Z*)‐3‐hexenol and thymol‐triggered calcium dynamics into physiological adaptations that bolster cold resistance. This discovery opens avenues for targeted genetic manipulation to enhance stress resilience in tea and other economically important crops.

Third, we demonstrated that the cold‐induced volatiles (*Z*)‐3‐hexenol and thymol significantly enhance cold tolerance in tea plants by triggering cytosolic Ca^2+^ elevation. Other volatiles, including eugenol, geraniol, and MeSA, also induced Ca^2+^ influx in tea protoplasts and have been associated with improved cold resistance (Zhao, Wang, et al. 2020; Zhao et al. 2022), further supporting a role for calcium signalling in VOC‐mediated responses. In contrast, 1‐hexanol and hexyl acetate, which did not elicit detectable Ca^2+^ signals, failed to enhance cold tolerance, underscoring a strong correlation between VOC‐induced Ca^2+^ signalling and cold acclimation. It is worth noting that (*Z*)‐3‐hexenol and thymol elicited stronger Ca^2+^ transients than eugenol, geraniol, and MeSA in our assays, suggesting that these VOCs may exert a more potent protective effect against cold damage, warranting further investigation. Nonetheless, we acknowledge that not all cold‐induced VOCs triggered measurable Ca^2+^ signals, suggesting that some may operate through Ca^2+^‐independent mechanisms. Alternative pathways, such as ROS accumulation, mitogen‐activated protein kinase (MAPK) cascades, or hormonal signalling via abscisic acid (ABA) and jasmonic acid (JA), have been implicated in plant stress responses and may be activated by specific VOCs (Zhao, Zhang, et al. 2020; Zhao et al. 2022; Chen et al. 2023; Jiang et al. 2023; Payá et al. 2023). A deeper understanding of these distinct or converging mechanisms could offer broader insights into VOC‐mediated stress adaptation in tea plants and other species.

Finally, while limited studies have reported on calcium fluorescence staining in living cells (Yang et al. 2023), we established a Fluo‐8‐based calcium detection system that enables real‐time monitoring of cytoplasmic calcium dynamics in tea protoplasts. This system proved highly effective in distinguishing functional VOCs based on their ability to trigger specific [Ca^2+^]_cyt_ responses, providing a robust platform for investigating VOC signalling. By overcoming the limitations of traditional fluorescence protein‐based calcium sensors (Aratani et al. 2023), this method makes calcium imaging accessible to non‐transformable plants like tea. Similar approaches using alternative fluorescent probes, such as fluo‐8/AM, fluo‐4/AM, and Rhod‐2/AM, have been employed to detect cytosolic calcium in various plant species, including apple ( *Malus domestica* ) (Qiu et al. 2020), wheat (Lindberg et al. 2012), *Pyrus pyrifolia* (Qu et al. 2016) and other plants (Suwińska et al. 2017). In our study, we opted for Fluo‐8/AM due to its higher sensitivity compared to its predecessor, Fluo‐3/AM and Fluo‐4/AM. Fluo‐8/AM efficiently enters protoplasts, allowing us to accurately detect calcium fluorescence in resting cells, optimise treatment concentrations, and monitor the real‐time calcium fluctuations triggered by environmental factors and volatile compounds. Using this system, we systematically screened for cold‐induced VOCs and identify novel compounds, (*Z*)‐3‐hexenol and thymol, that strongly enhance cold tolerance. Beyond VOCs, the system also detected calcium signalling responses to cold, hyper‐osmotic, and salt stress, suggesting its potential application in identifying other stress factors that trigger calcium signalling (Jiang et al. 2019; Laohavisit et al. 2020; Wu et al. 2020). However, this system relies on protoplast isolation, a process that may alter cellular physiology and potentially influence calcium signalling responses. Future advancements should aim to develop methods for tissue‐wide or systemic calcium imaging in intact plants to better replicate in vivo conditions. Such improvements could extend the utility of this system to broader applications in plant stress biology.

**FIGURE 7:**
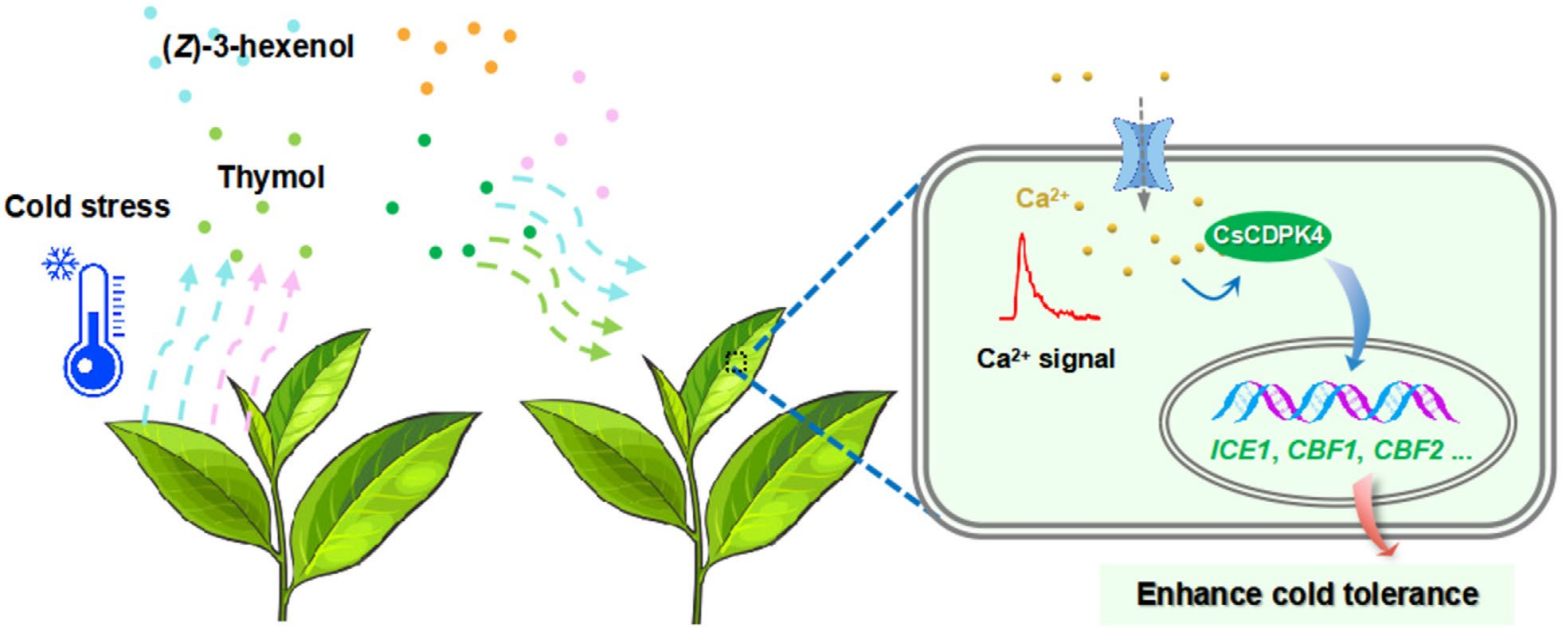
Proposed model illustrating how cold‐induced (*Z*)‐3‐hexenol and thymol enhance cold tolerance in tea plants. Cold‐induced volatile organic compounds (VOCs), such as (*Z*)‐3‐hexenol and thymol, are perceived by tea plants and trigger increases in cytosolic Ca^2+^ concentration ([Ca^2+^]_cyt_). This calcium signal activates the Ca^2+^ sensor protein CsCDPK4, which in turn induces the expression of cold‐responsive genes, including *CsICE1*, *CsCBF1*, and *CsCBF2*, and enhances the activities of antioxidant enzymes such as SOD and POD. Together, these responses contribute to improved cold tolerance in tea plants.

In summary, tea plants release specific VOC blends under cold stress, and some of these VOCs, such as (*Z*)‐3‐hexenol and thymol, activate cellular calcium signalling, including [Ca^2+^]_cyt_ fluctuations and *CsCDPK4* expression. This activation enhances the expression of cold stress‐related genes (*CsICE1*, *CsCBF1*, and *CsCBF2*) and promotes the activities of protective antioxidant enzymes (SOD and POD), resulting in increased tolerance to cold stress (Figure 7). Our findings not only advance the understanding of cold‐induced VOCs, but also provide a mechanistic framework linking VOC perception, calcium signalling, and stress tolerance. Integrating chemical ecology with calcium signalling offers novel insights into plant adaptation strategies and presents promising opportunities for improving agricultural sustainability in the face of climate change.

## 4 Materials and Methods

### 4.1 Plant Materials

Tender shoots of tea plants (*C. sinensis var. sinensis cv*. ‘shuchazao’) at least five years old were collected from the Nongcuiyuan tea plantation of Anhui Agriculture University (Hefei, China). For protoplast isolation, the complete, healthy, and tender 2nd leaf (2 L) and 3rd leaf (3 L) blades (Figure S4) were picked and surface sterilised with 75% alcohol for the following experiments. Plant samples with one bud and two leaves were collected to measure cold stress resistance.

### 4.2 VOCs Exposure and Cold Stress Treatment in Tea Plants

#### 4.2.1 VOCs Exposure Experiment

To investigate the regulatory role of cold‐induced VOCs in cold tolerance, a full VOC blend exposure experiment was conducted (Figure S1). Healthy tea plants were subjected to −5°C for 2 h and then placed in a 5 L glass vessel. Tea plants grown under normal conditions served as controls. Volatiles released by cold‐stressed or normal‐growth tea plants were pumped into adjacent glass vessels containing healthy tea plants for an 8‐h exposure period. Following the exposure treatment, the tea plants were either subjected to an additional 2‐h treatment at −5°C or maintained under normal conditions without cold stress. Then young tissues with one bud and two leaves were harvested for antioxidant activity assays and gene expression analysis.

#### 4.2.2 Specific VOC Exposure Experiment

To evaluate the ability of specific VOCs to enhance cold stress tolerance, tea plants were exposed to these compounds at concentrations corresponding to those naturally released by tea plants under cold stress (detailed calculations provided in the Section 4.3). Solutions of commercially available specific VOCs were applied to cotton pieces placed in 5 L glass vessels containing healthy tea plants for 8 h. Each VOC was dissolved in dimethyl sulfoxide (DMSO), and neat DMSO was used as the control. After exposure, the tea plants were either subjected to a 2‐h treatment at −5°C or maintained under normal conditions without cold stress. Following the treatment, leaves were harvested for antioxidant activity assays and gene expression analysis. Commercial volatile standards were purchased from Sigma‐Aldrich.

### 4.3 VOCs Quantification

Volatiles emitted from tea plants under cold stress were identified and quantified as described previously (Jing et al. 2019). Briefly, to detect volatiles induced by cold stress, tea plants were subjected to −5°C for 2 h, which were then placed in a large glass vessel (5 L) for 1 h. Then, volatiles were collected by SPME fibre for 60 min under controlled conditions (25°C ± 2°C, 70%–80% humidity). After collection, the SPME fibre with absorbed volatile metabolites was subjected to GC–MS analysis. Volatile compounds were thermally desorbed by inserting the SPME fibre into a GC injector connected to a fused silica GC column (60 m × 0.25 mm, film thickness 0.25 μm, J&W Scientific, Folsom, CA, USA) for 5 min. The injector temperature was 250°C using a splitless injection mode. Helium (> 99.99%) was used as the carrier gas at a flow rate of 1 mL/min. The oven program was set as follows: 35°C (2 min), 2°C/min to 60°C (3 min), 2°C/min to 110°C (2 min), and 10°C/min to 250°C (3 min). Electron‐impact mass spectra were generated at 70 eV, with a scan range from 30 to 600 *m*/*z*; the ion source temperature was 280°C, and the MS interface temperature was 300°C. Compounds were identified based on the mass‐spectral library NIST (National Institute of Standards and Technology).

To simulate the physiological concentrations of volatiles released by cold‐stressed tea plants, we collected data and calculated the release rates of different concentrations of pure volatile compounds. Individual volatile reagents at gradient concentrations were applied to cotton pieces, which were then placed in a 5 L glass vessel for 1 h. The released volatiles were quantified using GC–MS, and a standard curve was established based on the peak areas corresponding to the gradient concentrations (Figure S8). Based on the volatile release profiles of cold‐stressed tea plants (Table S1), 0.01 and 0.1 μM of specific VOCs were selected as physiological concentrations to simulate natural emissions for subsequent exposure treatments.

### 4.4 Ca^2+^ Inhibitor Treatments

To determine the function of calcium signalling, 10 mM LaCl_3_ (Lanthanum (III) chloride, a Ca^2+^‐channel blocker, Sigma) or 20 mM EGTA (glycol‐bis‐(2‐aminoethylether)‐N,N,N′,N′‐tetraacetic acid, chelate calcium, Sigma) was used to pre‐treat the tea plants (Knight et al. 1996; Aratani et al. 2023). All inhibitors were prepared in distilled water containing 0.02% (v/v) Tween‐20. To inhibit Ca^2+^ signalling, tea plants were evenly sprayed with LaCl_3_ or EGTA for 2 h, and then exposed to volatiles for 8 h. After exposure, the tea seedlings were either exposed to a 2‐h treatment at −5°C or maintained under normal temperature conditions. To evaluate the side effect of LaCl_3_ and EGTA alone, a separate set of plants was treated with the inhibitors using the same protocol and then either exposed to −5°C or kept under normal conditions without volatile application.

To examine the effects of calcium inhibitors on cellular Ca^2+^ dynamics in tea protoplasts, Fluo‐8 loaded cells were pre‐treated with 10 mM LaCl_3_ or 20 mM EGTA for 5 min prior to the application of stress treatments (cold, salt, or hyper‐osmotic stress) or VOC exposure.

### 4.5 Measurement of Fv/Fm, SOD and POD Activities

The net photosynthetic rate and maximum efficiency of photosystem II photochemistry (Fv/Fm) was measured using a pulse‐modulated fluorimeter Imaging‐PAM (Walz, Effeltrich, Germany). Cold stress‐treated tea plants were recovered for 30 min in the dark, then the Fv/Fm was measured. Fv/Fm was quantified using Imaging WinGegE software.

The activities of SOD and POD were measured as described (Zhao, Zhang, et al. 2020). Briefly, 0.2 g of leaf samples were homogenised in 2 mL cold extraction buffer (0.1 M phosphate buffer, pH 7.0). After centrifugation at 8000 rpm for 10 min, the supernatants were analysed using the Superoxide Dismutase (SOD) assay kit (WST‐1 method) and the Peroxidase assay kit (Nanjing Jiancheng Bioengineering Institute, Nanjing, China).

### 4.6 Tea Leaf Protoplasts Isolation

Leaf protoplasts were isolated with modifications based on previously described protocols (Yoo et al. 2007; Wang et al. 2022). The enzyme solution was prepared using the following components: 0.4 M D‐mannitol (Sigma, Germany), 0.5% Macerozyme R10 (Yakult, Tokyo, Japan), 0.7% Snailase (YEASEN, Shanghai, China), and 1.5% Cellulase R10 (Yakult, Japan), supplemented with 20 mM 4‐morpholineethanesulfonic acid (MES, pH = 5.7), 20 mM KCl (Sigma), 10 mM CaCl_2_ (Sigma), 10 mM β‐mercaptoethanol (Macklin, Shanghai, China), and 0.1% bovine serum albumin (BSA; Sigma). The final 20 mL enzyme solution was sterilised using a 0.45 μm syringe filter and dispensed into sterile glass Petri dishes (90 mm inner diameter). Fresh tea leaves (~0.5 g) were sliced into thin strips, with the major veins and leaf margins removed, and immediately transferred into the enzyme solution. Samples were vacuum‐infiltrated for 5 min to facilitate enzyme penetration, followed by static incubation at 25°C in the dark for 6 h. Gentle manual shaking was performed once every hour to enhance digestion efficiency. After digestion, the cell suspension was filtered through a 40 μm nylon mesh to remove undigested tissue. The resulting protoplast suspension was washed twice with 0.4 M D‐mannitol by gentle centrifugation and finally resuspended in 0.4 M D‐mannitol supplemented with 1 mM MES/KOH (pH 5.7). All centrifugation steps were conducted using a swinging bucket rotor (3‐18KS, Sigma, Germany) at 100 × *g* with acceleration and deceleration settings of 2, minimising shear stress and maximising cell viability. Isolated protoplasts were allowed to recover at 22°C for4 to 66 h before further use.

Protoplast quality was evaluated using two main criteria: cell viability and debris proportion. Viability was assessed by fluorescein diacetate (FDA) staining (2 μg/mL), with FDA‐positive cells considered viable. The percentage of viable cells was calculated as the number of FDA‐positive cells divided by the total number of cells. Observations were made under a confocal laser scanning microscope (Leica DMi8, Germany). Debris content was quantified using a haemocytometer by calculating the ratio of non‐cellular debris particles to total counted particles in a 100 μL aliquot. Only protoplast preparations with > 90% viability and ≤ 10% debris content were considered qualified and used for downstream analyses.

### 4.7 Imaging of [Ca^2+^]_cyt_ in Tea Leaf Protoplasts

To image cytosolic calcium concentration ([Ca^2+^]_cyt_) in tea leaf protoplasts, previously published methods were followed with minor adjustments (Qiu et al. 2020). Freshly isolated protoplasts were suspended in 0.4 M D‐mannitol containing 1 mM MES and incubated at 22°C under moderate light conditions (~100 μmol m^−2^ s^−1^). For calcium imaging, the protoplasts were then loaded with the Ca^2+^ sensitive fluorescent dye Fluo‐8/AM (ATT Bioquest, USA, hereafter referred to as Fluo‐8). To optimise dye loading and ensure cell viability, a range of Fluo‐8 concentrations (0.5–50 μM) was tested. A final concentration of 5 μM, with incubation at 37°C for 30 min in the dark, was determined to yield the best results, providing strong signal intensity with minimal cytotoxicity or background fluorescence (Figure 2a,b). After loading, excess dye was removed by gently washing the cells three times with the same D‐mannitol‐based buffer. The protoplasts were then transferred to a confocal imaging plate and incubated at 22°C for 2 h to allow recovery and stabilisation of basal [Ca^2+^]_cyt_ levels. Fluorescence imaging was performed using a laser scanning confocal microscope (Leica DMi8, Germany), with excitation at 488 nm and emission collected between 520 and 5500 nm. Calcium dynamics were quantified by normalising the fluorescence intensity at each time point (*F*) to the baseline fluorescence (*F*_0_), and relative change in fluorescence was calculated as (*F*–*F*_0_)/*F*_0_. All fluorescence intensity data were analysed using ImageJ software (https://imagej.net/ij/index.html).

### 4.8 Real‐Time Detection of [Ca^2+^]_cyt_ in Tea Leaf Protoplasts

Tea leaf protoplasts were suspended in 0.4 M D‐mannitol solution containing 1 mM MES and incubated with Fluo‐8 dye for 30 min at 37°C in the dark for calcium fluorescence labeling. Two hours after dye loading, the protoplasts were transferred into a white 96‐well plate. Solutions containing different abiotic stressors or volatile compounds were automatically injected into the wells. During treatment, the fluorescence intensity of [Ca^2+^]_cyt_ was recorded at one‐second intervals (for abiotic stressors) or 1‐min intervals (for volatile compounds) using a multimode microplate reader (Promega, USA). Relative fluorescence intensity was calculated as described above.

### 4.9 Protoplast Treatments

For cold stress treatment, Fluo‐8‐loaded cells were incubated in culture buffer pre‐cooled to 0°C for varying durations, while buffer at room temperature served as the control. For salt stress, NaCl solution was added to the protoplast suspension to achieve a final concentration of 0.5 M. For hyper‐osmotic stress, sorbitol was added to reach a final concentration of 1 M. For volatile treatments, commercial volatile standards were added to reach a final concentration of 0.01 μM, using 0.1% DMSO as the co‐solvent. The corresponding control group was treated with 0.1% DMSO alone. Following these treatments, protoplasts were either subjected to real‐time calcium signal detection using a multimode microplate reader or imaged using a confocal laser scanning microscope to visualise [Ca^2+^]_cyt_ dynamics.

### 4.10 Screening of Cold‐Induced Volatiles in Protoplasts

The dynamic changes of [Ca^2+^]_cyt_ in leaf protoplasts induced by volatiles were detected according to the method previously described with modifications (Laohavisit et al. 2020). Protoplast cells (200 μL) loaded with Fluo‐8 were cultured in vitro in a white 96‐well plate. Solutions containing volatiles (100 μL) were automatically injected into the wells of a white 96‐well plate at final concentrations of 0.01 and 0.1 μM. The fluorescence intensity of calcium was recorded at intervals of 1 min using the multimode microplate reader (Promega, USA). 0.1% DMSO‐treated cells were used as control. Before volatile solutions treatment, the background calcium fluorescence intensity was collected.

### 4.11 Quantitative Real‐Time PCR Analysis

RNA was isolated from leaves of tea plants using FastPure Universal Plant Total RNA Isolation Kit (Vazyme, China). Reverse transcription of cDNA from total RNA was performed with Hifair V one‐step RT‐gDNA digestion SuperMix for qPCR Kit (Yeasen, China). Hieff SYBRGreen Master Mix (Yeasen, China) was used for quantifying the relative expression of specific genes. Actin family protein (CsACTIN) was used as an internal reference gene, and the relative expression was calculated using the 2^−∆∆CT^ method (Livak and Schmittgen 2001). The primers used for tested genes are listed in Table S3.

### 4.12 Gene Suppression of *CsCDPK4*

Candidate gene‐specific antisense oligonucleotides (asODNs) targeting *CsCDPK4* were designed using the Soligo software based on its cDNA sequence (Zhao, Zhang, et al. 2020; Zhao et al. 2022). *CsCDPK4* asODN primer: CTTGGCCTAGCTTTTTGCCA. A 1 mL solution containing 40 μM of the asODN was infiltrated into tea plant leaves. Plants treated with the corresponding sense oligonucleotides (sODNs) served as controls. Prior to subsequent analyses, the silencing efficiency of *CsCDPK4* and the expression levels of its homologous genes in the asODN‐treated plants were verified. After a 24‐h incubation, the plants were subjected to volatile exposure and cold tolerance assays.

### 4.13 Statistical Analysis

Independent experiments were performed at least three times. Statistical analysis was performed using EXCEL software (Microsoft). Data were presented as mean ± standard error (SE) or standard deviation (SD). *p* values < 0.05 were considered statistically significant. Two‐way analysis of variance (ANOVA) and Tukey honestly significant difference (HSD) post hoc test were used for data analysis.

### 4.14 Accession Numbers

Sequence data from this article can be found in the *C. sinensis* genome database (Tea Plant Information Archive [TPIA]: a comprehensive knowledge database for tea plant [teaplants.cn]) under accession numbers: CsCDPK4 (CSS0042249), CsCDPK17 (CSS0042985), CsCDPK10 (CSS0023979), CsCDPK28 (CSS0008031), CsCDPK30 (CSS0003317), CsCDPK1 (CSS0012731), CsCDPK2 (CSS0030469), and CsCDPK1L (CSS0006250).

## Author Contributions

Y.L., M.Y. and C.S. conceptualised the initial study; Y.L. and M.Y. designed the experimental layout; Y.L., Y.S., Z.L. and L.W. performed the lab experiments; Y.L. and M.Y. drafted the initial article; all authors discussed the results; Q.W., W.S., M.Y. and C.S. reviewed the manuscript and approved the final article.

## Conflicts of Interest

The authors declare no conflicts of interest.

## Supporting information


**Figure S1:** Experimental design for VOC exposure‐mediated cold tolerance in tea plants.
**Figure S2:** Calcium inhibitors LaCl_3_ and EGTA did not adversely affect key physiological parameters in tea plants.
**Figure S3:** Calcium signalling is likely involved in mediating VOC‐mediated cold tolerance in tea plants.
**Figure S4:** Isolation of tea plant protoplasts for cellular Ca^2+^ dynamics detection.
**Figure S5:** Effects of calcium inhibitors on cellular Ca^2+^ dynamics in tea cells using Fluo‐8 dye under different stress conditions.
**Figure S6:** High‐throughput screening of [Ca^2+^]_cyt_‐responsive volatiles induced by cold stress in tea protoplasts.
**Figure S7:** Effects of calcium inhibitors on (*Z*)‐3‐hexenol‐ and thymol‐induced [Ca^2+^]_cyt_ responses in tea protoplasts.
**Figure S8:** Standard curve of 1‐hexanol, hexyl acetate, (*Z*)‐3‐hexenol, and thymol.
**Figure S9:** [Ca^2+^]_cyt_‐responsive volatiles (*Z*)‐3‐hexenol and thymol prime the antioxidant system in the absence of cold stress.
**Figure S10:** Relative expression of *CsCDPK4* and its homologous genes in *CDPK4*‐silenced tea plants.
**Figure S11:** CsCDPK4 is essential for (*Z*)‐3‐hexenol and thymol‐induced cold stress tolerance in tea plants.
**Table S1:** Volatile compounds emitted from tea plants in the recovery phase after cold stress.
**Table S3:** Primers used for real‐time RT‐PCR assays.

**Table S2:** Expression patterns of calcium‐dependent protein kinases (*CDPKs*) in response to cold stress in tea plants.

## Data Availability

The data that supports the findings of this study are available in the Supporting Information of this article.

## References

[pbi70346-bib-0001] Adhikari, L. , R. Baral , D. Paudel , et al. 2022. “Cold Stress in Plants: Strategies to Improve Cold Tolerance in Forage Species.” Plant Stress 4: 100081.

[pbi70346-bib-0002] Allan, C. , R. J. Morris , C. N. Meisrimler , and K. J. Dietz . 2022. “Encoding, Transmission, Decoding, and Specificity of Calcium Signals in Plants.” Journal of Experimental Botany 73: 3372–3385. 35298633 10.1093/jxb/erac105 PMC9162177

[pbi70346-bib-0003] Aratani, Y. , T. Uemura , T. Hagihara , K. Matsui , and M. Toyota . 2023. “Green Leaf Volatile Sensory Calcium Transduction in Arabidopsis.” Nature Communications 14: 6236. 10.1038/s41467-023-41589-9 PMC10582025 37848440

[pbi70346-bib-0004] Asai, N. , T. Nishioka , J. Takabayashi , and T. Furuichi . 2009. “Plant Volatiles Regulate the Activities of Ca^2+^‐Permeable Channels and Promote Cytoplasmic Calcium Transients in Arabidopsis Leaf Cells.” Plant Signaling & Behavior 4: 294–300. 19794844 10.4161/psb.4.4.8275 PMC2664488

[pbi70346-bib-0005] Chen, Y. , Y. Cao , Y. Duan , et al. 2023. “The Effects of Overexpressing UDP‐Glycosyltransferases Genes on the Plant Response to Abiotic Stress: A Meta‐Analysis.” Beverage Plant Research 3: 28.

[pbi70346-bib-0006] Cheng, Y. , X. Kong , N. Wang , T. Wang , J. Chen , and Z. Q. Shi . 2020. “Thymol Confers Tolerance to Salt Stress by Activating Anti‐Oxidative Defense and Modulating Na^+^ Homeostasis in Rice Root.” Ecotoxicology and Environmental Safety 188: 109894. 31706239 10.1016/j.ecoenv.2019.109894

[pbi70346-bib-0007] Cofer, T. M. , M. Engelberth , and J. Engelberth . 2018. “Green Leaf Volatiles Protect Maize ( *Zea mays* ) Seedlings Against Damage From Cold Stress.” Plant, Cell & Environment 41: 1673–1682. 10.1111/pce.13204 29601632

[pbi70346-bib-0008] Ding, Y. , Y. Shi , and S. Yang . 2019. “Advances and Challenges in Uncovering Cold Tolerance Regulatory Mechanisms in Plants.” New Phytologist 222: 1690–1704. 30664232 10.1111/nph.15696

[pbi70346-bib-0009] Ding, Y. , H. Yang , S. Wu , et al. 2022. “CPK28‐NLP7 Module Integrates Cold‐Induced Ca^2+^ Signal and Transcriptional Reprogramming in Arabidopsis.” Science Advances 8: eabn7901. 35767615 10.1126/sciadv.abn7901 PMC9242591

[pbi70346-bib-0010] Engelberth, J. , H. T. Alborn , E. A. Schmelz , and J. H. Tumlinson . 2004. “Airborne Signals Prime Plants Against Insect Herbivore Attack.” Proceedings of the National Academy of Sciences of the United States of America 101: 1781–1785. 14749516 10.1073/pnas.0308037100 PMC341853

[pbi70346-bib-0011] Jiang, H. , M. Zhang , F. Yu , et al. 2023. “A Geraniol Synthase Regulates Plant Defense via Alternative Splicing in Tea Plants.” Horticulture Research 10: uhad184. 37885816 10.1093/hr/uhad184 PMC10599320

[pbi70346-bib-0012] Jiang, Z. , X. Zhou , M. Tao , et al. 2019. “Plant Cell‐Surface GIPC Sphingolipids Sense Salt to Trigger Ca^2+^ Influx.” Nature 572: 341–346. 31367039 10.1038/s41586-019-1449-z

[pbi70346-bib-0013] Jiao, C. , J. Gong , Z. Guo , S. Li , Y. Zuo , and Y. Shen . 2022. “Linalool Activates Oxidative and Calcium Burst and CAM3‐ACA8 Participates in Calcium Recovery in Arabidopsis Leaves.” International Journal of Molecular Sciences 23: 5357. 35628166 10.3390/ijms23105357 PMC9142083

[pbi70346-bib-0014] Jin, J. , M. Zhao , T. Jing , et al. 2023. “(Z)‐3‐Hexenol Integrates Drought and Cold Stress Signaling by Activating Abscisic Acid Glucosylation in Tea Plants.” Plant Physiology 193: 1491–1507. 37315209 10.1093/plphys/kiad346 PMC10517186

[pbi70346-bib-0015] Jin, J. , M. Zhao , T. Jing , et al. 2023. “Volatile Compound‐Mediated Plant–Plant Interactions Under Stress With the Tea Plant as a Model.” Horticulture Research 10: uhad143. 37691961 10.1093/hr/uhad143 PMC10483893

[pbi70346-bib-0016] Jing, T. , X. Qian , W. Du , et al. 2021. “Herbivore‐Induced Volatiles Influence Moth Preference by Increasing the Beta‐Ocimene Emission of Neighbouring Tea Plants.” Plant, Cell & Environment 44: 3667–3680. 10.1111/pce.14174 34449086

[pbi70346-bib-0017] Jing, T. , N. Zhang , T. Gao , et al. 2019. “Glucosylation of (*Z*)‐3‐Hexenol Informs Intraspecies Interactions in Plants: A Case Study in *Camellia sinensis* .” Plant, Cell & Environment 42: 1352–1367. 10.1111/pce.13479 30421786

[pbi70346-bib-0018] Kidokoro, S. , K. Shinozaki , and K. Yamaguchi‐Shinozaki . 2022. “Transcriptional Regulatory Network of Plant Cold‐Stress Responses.” Trends in Plant Science 27: 922–935. 35210165 10.1016/j.tplants.2022.01.008

[pbi70346-bib-0019] Knight, H. , A. J. Trewavas , and M. R. Knight . 1996. “Cold Calcium Signaling in Arabidopsis Involves Two Cellular Pools and a Change in Calcium Signature After Acclimation.” Plant Cell 8: 489–503. 8721751 10.1105/tpc.8.3.489 PMC161115

[pbi70346-bib-0020] Laohavisit, A. , T. Wakatake , N. Ishihama , et al. 2020. “Quinone Perception in Plants via Leucine‐Rich‐Repeat Receptor‐Like Kinases.” Nature 587: 92–97. 32879491 10.1038/s41586-020-2655-4

[pbi70346-bib-0021] Lindberg, S. , M. A. Kader , and V. Yemelyanov . 2012. “Calcium Signalling in Plant Cells Under Environmental Stress.” In Environmental Adaptations and Stress Tolerance of Plants in the Era of Climate Change, edited by P. Ahmad and M. N. V. Prasad , 325–360. Springer New York.

[pbi70346-bib-0022] Liu, Q. , Y. Ding , Y. Shi , et al. 2021. “The Calcium Transporter ANNEXIN1 Mediates Cold‐Induced Calcium Signaling and Freezing Tolerance in Plants.” EMBO Journal 40: e104559. 33372703 10.15252/embj.2020104559 PMC7809786

[pbi70346-bib-0023] Liu, Y. , C. Xu , Y. Zhu , et al. 2018. “The Calcium‐Dependent Kinase OsCPK24 Functions in Cold Stress Responses in Rice.” Journal of Integrative Plant Biology 60: 173–188. 29193704 10.1111/jipb.12614

[pbi70346-bib-0024] Livak, K. J. , and T. D. Schmittgen . 2001. “Analysis of Relative Gene Expression Data Using Real‐Time Quantitative PCR and the 2^−ΔΔCT^ Method.” Methods 25: 402–408. 11846609 10.1006/meth.2001.1262

[pbi70346-bib-0025] Luan, S. , and C. Wang . 2021. “Calcium Signaling Mechanisms Across Kingdoms.” Annual Review of Cell and Developmental Biology 37: 311–340. 10.1146/annurev-cellbio-120219-035210 34375534

[pbi70346-bib-0026] Ma, Y. , X. Dai , Y. Xu , et al. 2015. “COLD1 Confers Chilling Tolerance in Rice.” Cell 160: 1209–1221. 25728666 10.1016/j.cell.2015.01.046

[pbi70346-bib-0027] Mittler, R. , S. I. Zandalinas , Y. Fichman , and F. Van Breusegem . 2022. “Reactive Oxygen Species Signalling in Plant Stress Responses.” Nature Reviews Molecular Cell Biology 23: 663–679. 35760900 10.1038/s41580-022-00499-2

[pbi70346-bib-0028] Payá, C. , B. Belda‐Palazón , F. Vera‐Sirera , et al. 2023. “Signalling Mechanisms and Agricultural Applications of (*Z*)‐3‐Hexenyl Butyrate‐Mediated Stomatal Closure.” Horticulture Research 11: uhad248. 38239809 10.1093/hr/uhad248 PMC10794947

[pbi70346-bib-0029] Pei, S. , Y. Liu , W. Li , et al. 2022. “OSCA1 Is an Osmotic Specific Sensor: A Method to Distinguish Ca^2+^‐Mediated Osmotic and Ionic Perception.” New Phytologist 235: 1665–1678. 35527515 10.1111/nph.18217

[pbi70346-bib-0030] Qiu, L. , Y. Wang , and H. Qu . 2020. “Loading Calcium Fluorescent Probes Into Protoplasts to Detect Calcium in the Flesh Tissue Cells of *Malus domestica* .” Horticulture Research 7: 91. 32528703 10.1038/s41438-020-0315-3 PMC7261807

[pbi70346-bib-0031] Qu, H. , W. Xing , F. Wu , and Y. Wang . 2016. “Rapid and Inexpensive Method of Loading Fluorescent Dye Into Pollen Tubes and Root Hairs.” PLoS One 11: e0152320. 27055240 10.1371/journal.pone.0152320 PMC4824429

[pbi70346-bib-0032] Shi, Y. , Y. Ding , and S. Yang . 2018. “Molecular Regulation of CBF Signaling in Cold Acclimation.” Trends in Plant Science 23: 623–637. 29735429 10.1016/j.tplants.2018.04.002

[pbi70346-bib-0033] Smirnoff, N. , and D. Arnaud . 2019. “Hydrogen Peroxide Metabolism and Functions in Plants.” New Phytologist 221: 1197–1214. 30222198 10.1111/nph.15488

[pbi70346-bib-0034] Sugimoto, K. , E. Ono , T. Inaba , et al. 2023. “Identification of a Tomato UDP‐Arabinosyltransferase for Airborne Volatile Reception.” Nature Communications 14: 677. 10.1038/s41467-023-36381-8 PMC9908901 36755045

[pbi70346-bib-0035] Suwińska, A. , P. Wasąg , P. Zakrzewski , M. Lenartowska , and R. Lenartowski . 2017. “Calreticulin Is Required for Calcium Homeostasis and Proper Pollen Tube Tip Growth in Petunia.” Planta 245: 909–926. 28078426 10.1007/s00425-017-2649-0 PMC5391374

[pbi70346-bib-0036] Toyota, M. , D. Spencer , S. Sawai‐Toyota , et al. 2018. “Glutamate Triggers Long‐Distance, Calcium‐Based Plant Defense Signaling.” Science 361: 1112–1115. 30213912 10.1126/science.aat7744

[pbi70346-bib-0037] Upadhyay, R. , R. Saini , P. K. Shukla , and K. N. Tiwari . 2025. “Role of Secondary Metabolites in Plant Defense Mechanisms: A Molecular and Biotechnological Insights.” Phytochemistry Reviews 24: 953–983.

[pbi70346-bib-0038] Wang, B. , G. Zhou , Z. Xin , R. Ji , and Y. J. P. M. B. R. Lou . 2015. “(*Z*)‐3‐Hexenal, One of the Green Leaf Volatiles, Increases Susceptibility of Rice to the White‐Backed Planthopper *Sogatella furcifera* .” Plant Molecular Biology Reporter 33: 377–387.

[pbi70346-bib-0039] Wang, J. , Y. Hu , D. Guo , et al. 2024. “Evolution and Functional Divergence of Glycosyltransferase Genes Shaped the Quality and Cold Tolerance of Tea Plants.” Plant Cell 37: koae268. 39365921 10.1093/plcell/koae268 PMC11663605

[pbi70346-bib-0040] Wang, J. , Y. Ren , X. Liu , et al. 2021. “Transcriptional Activation and Phosphorylation of OsCNGC9 Confer Enhanced Chilling Tolerance in Rice.” Molecular Plant 14: 315–329. 33278597 10.1016/j.molp.2020.11.022

[pbi70346-bib-0041] Wang, Q. , Y. Wu , A. Peng , et al. 2022. “Single‐Cell Transcriptome Atlas Reveals Developmental Trajectories and a Novel Metabolic Pathway of Catechin Esters in Tea Leaves.” Plant Biotechnology Journal 20: 2089–2106. 35810348 10.1111/pbi.13891 PMC9616531

[pbi70346-bib-0042] Wu, F. , Y. Chi , Z. Jiang , et al. 2020. “Hydrogen Peroxide Sensor HPCA1 Is an LRR Receptor Kinase in Arabidopsis.” Nature 578: 577–581. 32076270 10.1038/s41586-020-2032-3

[pbi70346-bib-0043] Wu, J. , M. Nadeem , L. Galagedara , R. Thomas , and M. Cheema . 2022. “Recent Insights Into Cell Responses to Cold Stress in Plants: Signaling, Defence, and Potential Functions of Phosphatidic Acid.” Environmental and Experimental Botany 203: 105068.

[pbi70346-bib-0044] Yang, S. , W. Cai , L. Shen , et al. 2022. “A CaCDPK29‐CaWRKY27b Module Promotes CaWRKY40‐Mediated Thermotolerance and Immunity to *Ralstonia solanacearum* in Pepper.” New Phytologist 233: 1843–1863. 34854082 10.1111/nph.17891

[pbi70346-bib-0045] Yang, X. , Q. Zhang , S. Zhang , et al. 2023. “Molecule Fluorescent Probes for Sensing and Imaging Analytes in Plants: Developments and Challenges.” Coordination Chemistry Reviews 487: 215154.

[pbi70346-bib-0046] Ye, M. , M. Liu , M. Erb , et al. 2021. “Indole Primes Defence Signalling and Increases Herbivore Resistance in Tea Plants.” Plant, Cell & Environment 44: 1165–1177. 10.1111/pce.13897 32996129

[pbi70346-bib-0047] Ye, X. , T. Ling , Y. Xue , et al. 2016. “Thymol Mitigates Cadmium Stress by Regulating Glutathione Levels and Reactive Oxygen Species Homeostasis in Tobacco Seedlings.” Molecules 21: 1339. 27754435 10.3390/molecules21101339 PMC6273743

[pbi70346-bib-0048] Yip Delormel, T. , and M. Boudsocq . 2019. “Properties and Functions of Calcium‐Dependent Protein Kinases and Their Relatives in *Arabidopsis thaliana* .” New Phytologist 224: 585–604. 31369160 10.1111/nph.16088

[pbi70346-bib-0049] Yoo, S. D. , Y. H. Cho , and J. Sheen . 2007. “Arabidopsis Mesophyll Protoplasts: A Versatile Cell System for Transient Gene Expression Analysis.” Nature Protocols 2: 1565–1572. 17585298 10.1038/nprot.2007.199

[pbi70346-bib-0050] Yuan, F. , H. Yang , Y. Xue , et al. 2014. “OSCA1 Mediates Osmotic‐Stress‐Evoked Ca^2+^ Increases Vital for Osmosensing in Arabidopsis.” Nature 514: 367–371. 25162526 10.1038/nature13593

[pbi70346-bib-0051] Zebelo, S. A. , K. Matsui , R. Ozawa , and M. E. Maffei . 2012. “Plasma Membrane Potential Depolarization and Cytosolic Calcium Flux Are Early Events Involved in Tomato (*Solanum lycopersicon*) Plant‐To‐Plant Communication.” Plant Science 196: 93–100. 23017903 10.1016/j.plantsci.2012.08.006

[pbi70346-bib-0052] Zhang, H. , J. Zhu , Z. Gong , and J. Zhu . 2021. “Abiotic Stress Responses in Plants.” Nature Reviews Genetics 23: 104–119. 10.1038/s41576-021-00413-0 34561623

[pbi70346-bib-0053] Zhao, M. , J. Jin , J. Wang , et al. 2022. “Eugenol Functions as a Signal Mediating Cold and Drought Tolerance via UGT71A59‐Mediated Glucosylation in Tea Plants.” Plant Journal 109: 1489–1506. 10.1111/tpj.15647 34931743

[pbi70346-bib-0054] Zhao, M. , L. Wang , J. Wang , et al. 2020. “Induction of Priming by Cold Stress via Inducible Volatile Cues in Neighboring Tea Plants.” Journal of Integrative Plant Biology 62: 1461–1468. 32275096 10.1111/jipb.12937

[pbi70346-bib-0055] Zhao, M. , N. Zhang , T. Gao , et al. 2020. “Sesquiterpene Glucosylation Mediated by Glucosyltransferase UGT91Q2 Is Involved in the Modulation of Cold Stress Tolerance in Tea Plants.” New Phytologist 226: 362–372. 31828806 10.1111/nph.16364

