# Supplementary material for: Cold Stress‐Induced (Z)‐3‐Hexenol and Thymol Enhance Cold Tolerance of Tea Plants by Activating Ca2+ Signalling
Source: Plant Biotechnol J. 2025 Sep 3;23(12):5833–48. doi: 10.1111/pbi.70346 (PMC12665057; doi:10.1111/pbi.70346)
Supplement: Supplementary file 1 — Figure S1: Experimental design for VOC exposure‐mediated cold tolerance in tea plants. Figure S2: Calcium inhibitors LaCl3 and EGTA did not adversely affect key physiological parameters in tea plants. Figure S3: Calcium signalling is likely involved in mediating VOC‐mediated cold tolerance in tea plants. Figure S4: Isolation of tea plant protoplasts for cellular Ca2+ dynamics detection. Figure S5: Effects of calcium inhibitors on cellular Ca2+ dynamics in tea cells using Fluo‐8 dye under different stress conditions. Figure S6: High‐throughput screening of [Ca2+]cyt‐responsive volatiles induced by cold stress in tea protoplasts. Figure S7: Effects of calcium inhibitors on (Z)‐3‐hexenol‐ and thymol‐induced [Ca2+]cyt responses in tea protoplasts. Figure S8: Standard curve of 1‐hexanol, hexyl acetate, (Z)‐3‐hexenol, and thymol. Figure S9: [Ca2+]cyt‐responsive volatiles (Z)‐3‐hexenol and thymol prime the antioxidant system in the absence of cold stress. Figure S10: Relative expression of CsCDPK4 and its homologous genes in CDPK4‐silenced tea plants. Figure S11: CsCDPK4 is essential for (Z)‐3‐hexenol and thymol‐induced cold stress tolerance in tea plants. Table S1: Volatile compounds emitted from tea plants in the recovery phase after cold stress. Table S3: Primers used for real‐time RT‐PCR assays. [file PBI-23-5833-s002.pdf]

Supporting Information

Title: Cold stress-induced (*Z*)-3-hexenol and thymol enhance cold tolerance of tea plants by activating Ca^{2+} signaling

Authors: Yuantao Liu[1, 2], Yaling Song[1], Zhengwei Luo[1], Lisha Wang[1], Jieyang Jin[1], Tingting Jing[1], Mingyue Zhao[1], Qiang Wang[1], Wilfried Schwab[1, 3], Meng Ye[2]*, Chuankui Song[1]*

Author for correspondence:

*Corresponding author: Chuankui Song; Meng Ye

E-mail: sckfriend@163.com; yemeng@caas.cn

The following Supporting Information is available for this article:

Fig. S1 Experimental design for VOC exposure-mediated cold tolerance in tea plants

Fig. S2 Calcium inhibitors $LaCl_3$ and EGTA did not adversely affect key physiological parameters in tea plants

Fig. S3 Calcium signaling is likely involved in mediating VOC-mediated cold tolerance in tea plants

Fig. S4 Isolation of tea plant protoplasts for cellular Ca^{2+} dynamics detection

Fig. S5 Effects of calcium inhibitors on cellular Ca^{2+} dynamics in tea cells using Fluo-8 dye under different stress conditions

Fig. S6 High-throughput screening of $[Ca^{2+}]_{cyt}$-responsive volatiles induced by cold stress in tea protoplasts

Fig. S7 Effects of calcium inhibitors on (*Z*)-3-hexenol- and thymol-induced $[Ca^{2+}]_{cyt}$ responses in tea protoplasts

Fig. S8 Standard curve of 1-hexanol, hexyl acetate, (*Z*)-3-hexenol, and thymol

Fig. S9 $[Ca^{2+}]_{cyt}$-responsive volatiles (*Z*)-3-hexenol and thymol prime the antioxidant system in the absence of cold stress

Fig. S10 Relative expression of *CsCDPK4* and its homologous genes in *CDPK4*-silenced tea plants

Fig. S11 CsCDPK4 is essential for (*Z*)-3-hexenol and thymol-induced cold stress tolerance in tea plants

Table S1 Volatile compounds emitted from tea plants in the recovery phase after cold stress

Table S2 Expression patterns of calcium-dependent protein kinases (*CDPKs*) in response to cold stress in tea plants

Table S3 Primers used for real time RT-PCR assays

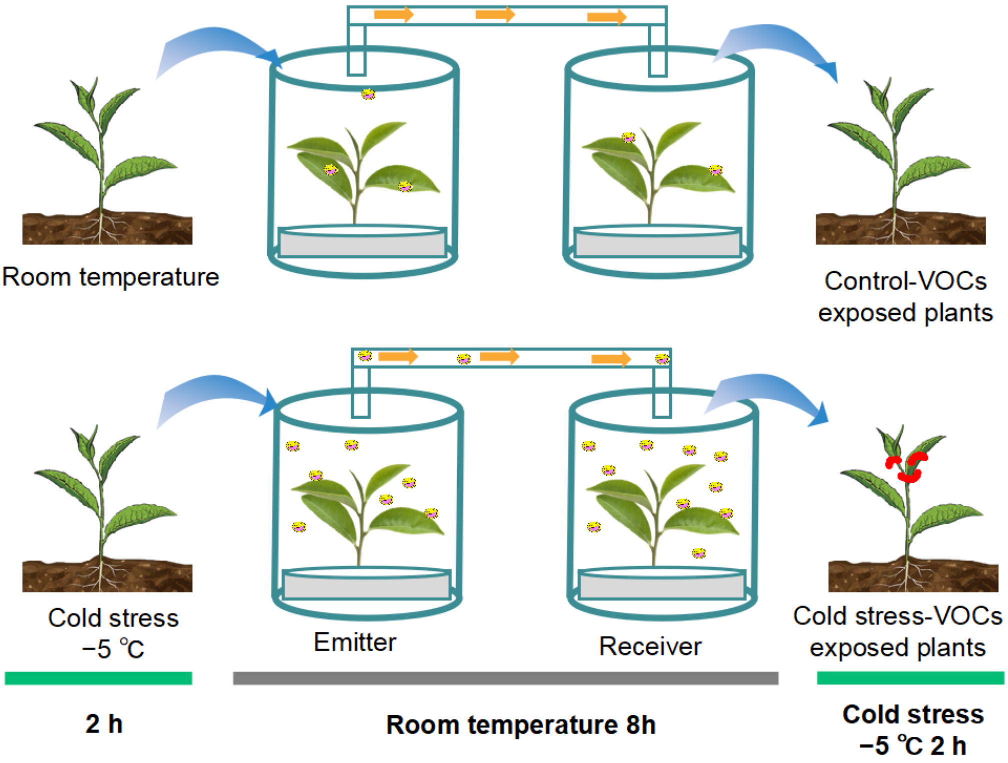

Fig. S1 Experimental design for VOC exposure-mediated cold tolerance in tea plants

The healthy tea plants were subjected to -5 ℃ for 2 h to induce VOC release, while unstressed plants under normal growth conditions served as controls. The volatiles released by stressed or unstressed tea plants were pumped into adjacent 5 L glass vessels containing healthy tea plants for 8 hours of exposure. Following VOC exposure, the treated tea plants were subjected to -5°C for 2 hours. Physiological measurements were then performed to assess the cold stress responses of the tea plants.

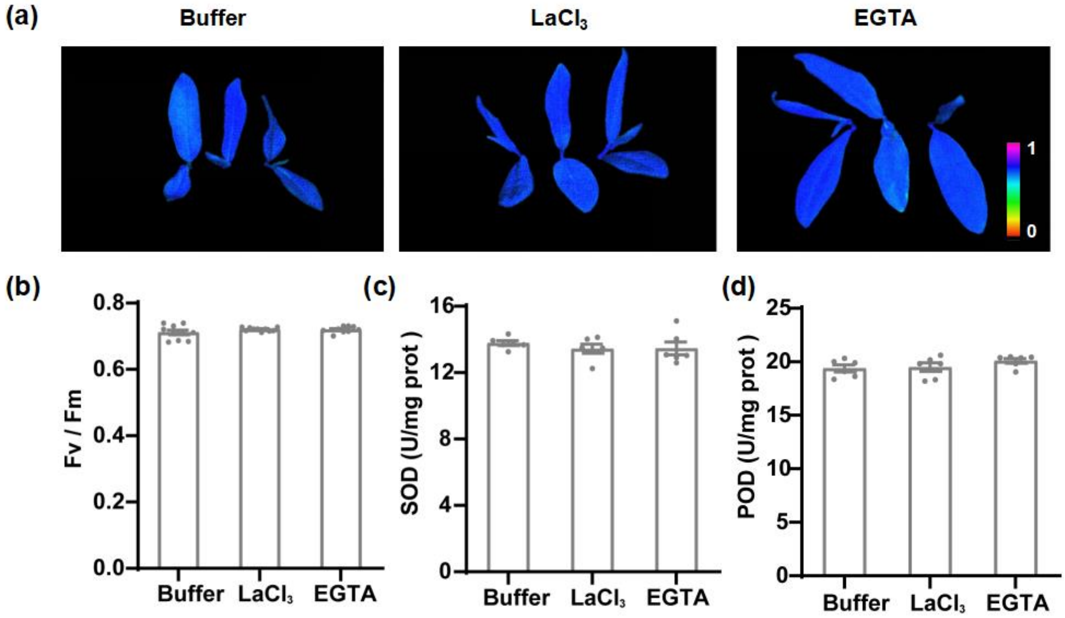

Fig. S2 Calcium inhibitors $LaCl_3$ and EGTA did not adversely affect key physiological parameters in tea plants

(a) Chlorophyll fluorescence images of tea plants treated with $LaCl_3$ or EGTA alone, without cold stress treatment. Fv/Fm is presented using a pseudo-color scale ranging from 0 to 1. **(b–d)** Measurements of Fv/Fm (b), SOD activity (c), and POD activity (d) in tea plants treated with $LaCl_3$ or EGTA (± SE; n = 10 plants for b, n = 6 plants for c and d). Data points represent individual replicates.

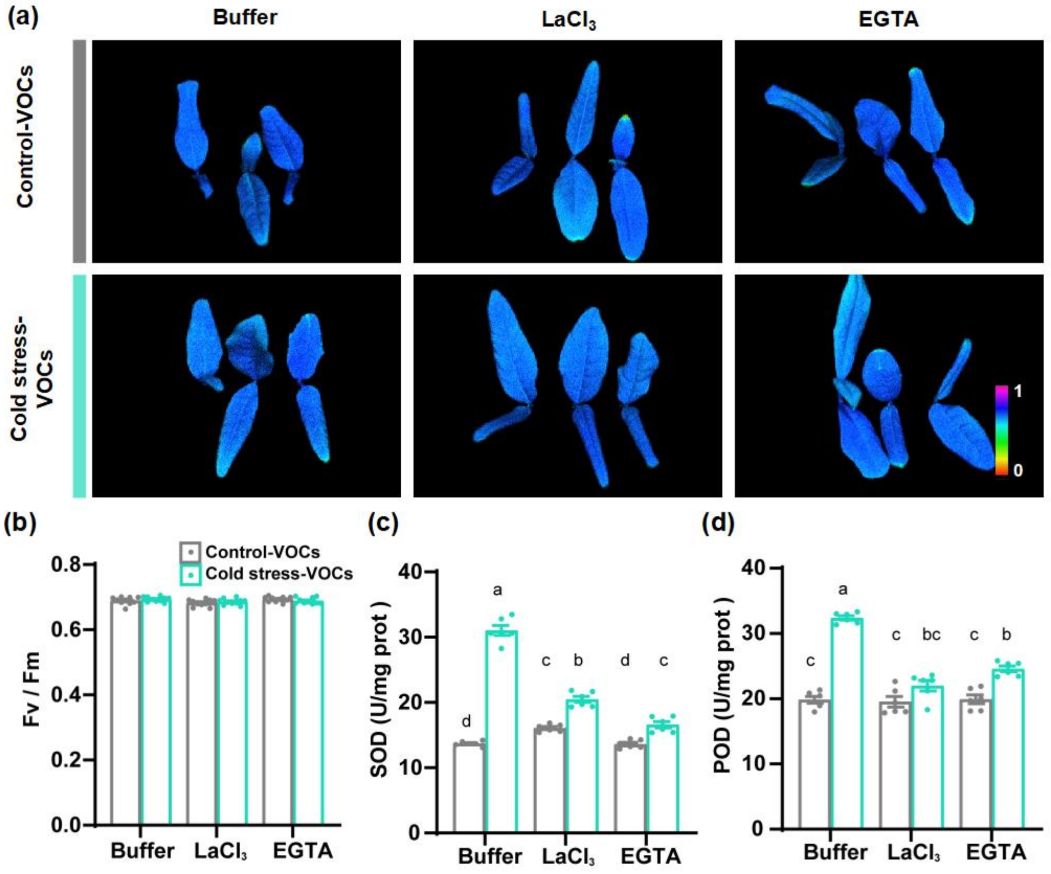

Fig. S3 Calcium signaling is likely involved in mediating VOC-induced cold tolerance in tea plants

(a) Chlorophyll fluorescence images of tea plants pre-treated with calcium inhibitors $LaCl_3$ or EGTA, then exposed to VOCs emitted from either control or cold-stressed plants in ambient conditions. Fv/Fm is presented using a pseudo-color scale ranging from 0 to 1. **(b–d)** Measurements of Fv/Fm (b), SOD activity (c), and POD activity (d) in tea plants pre-treated with $LaCl_3$ or EGTA, then exposed to VOCs emitted from either control or cold-stressed plants in ambient conditions (± SE; n = 10 plants for b, n = 6 plants for c and d). Data points represent individual replicates. Different letters indicate significant differences at $P < 0.05$ (two-way ANOVA with Tukey's HSD post hoc test).

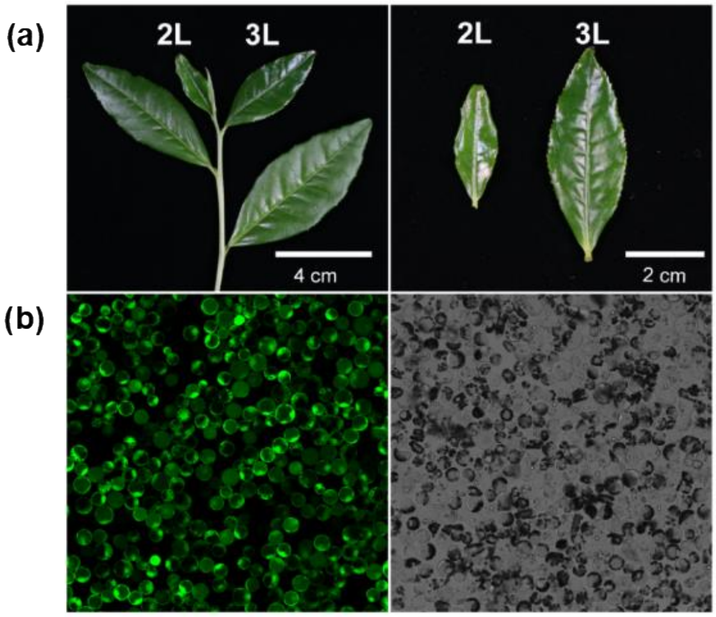

Fig. S4 Isolation of tea plant protoplasts for cellular Ca^{2+} dynamics detection

(a) The 2nd and 3rd leaves of young shoot of *C. sinensis var.* ‘Shuchazao’, represented as 2 L and 3 L, respectively, were used for protoplast extraction. **(b)** Protoplasts were stained with or without fluorescein diacetate (FDA). Viable protoplasts exhibit a rounded morphology and emit green fluorescence after FDA staining.

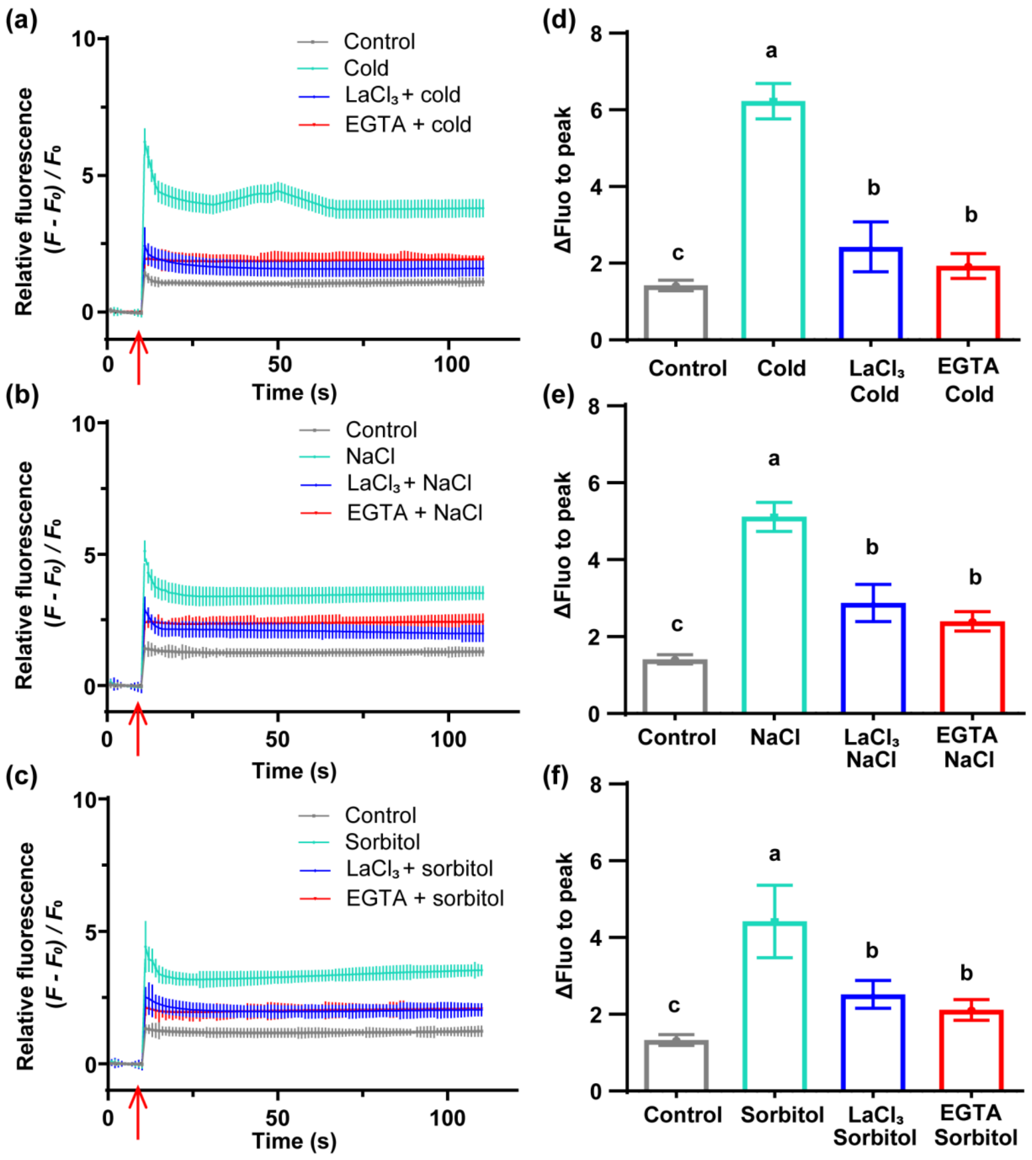

Fig. S5 Effects of calcium inhibitors on cellular Ca^{2+} dynamics in tea leaf cells using Fluo-8 dye under different stress conditions

(a-c) Time-course quantification of $[Ca^{2+}]_{cyt}$ dynamics in tea protoplasts subjected to cold (0°C; a), salt (0.5 M NaCl; b), and hyper-osmotic stress (1 M sorbitol; c), with or without pre-treatment with calcium inhibitors. Luminescence was recorded at 1-second intervals using a multimode microplate reader (± SE; *n* = 8 replicates). The red arrow indicates the time point of reagent addition and reaction initiation. The relative fluorescence intensity, calculated as $(F - F_0) / F_0$, was determined by normalizing the fluorescence intensity at each time point (F) to the baseline fluorescence intensity (F_0). **(d-f)** Comparison of the maximal Ca^{2+} fluorescence intensity changes in protoplasts under each condition as presented in (a-c). Different letters indicate statistically significant differences at $P < 0.05$ (two-way ANOVA with Tukey's HSD post hoc test).

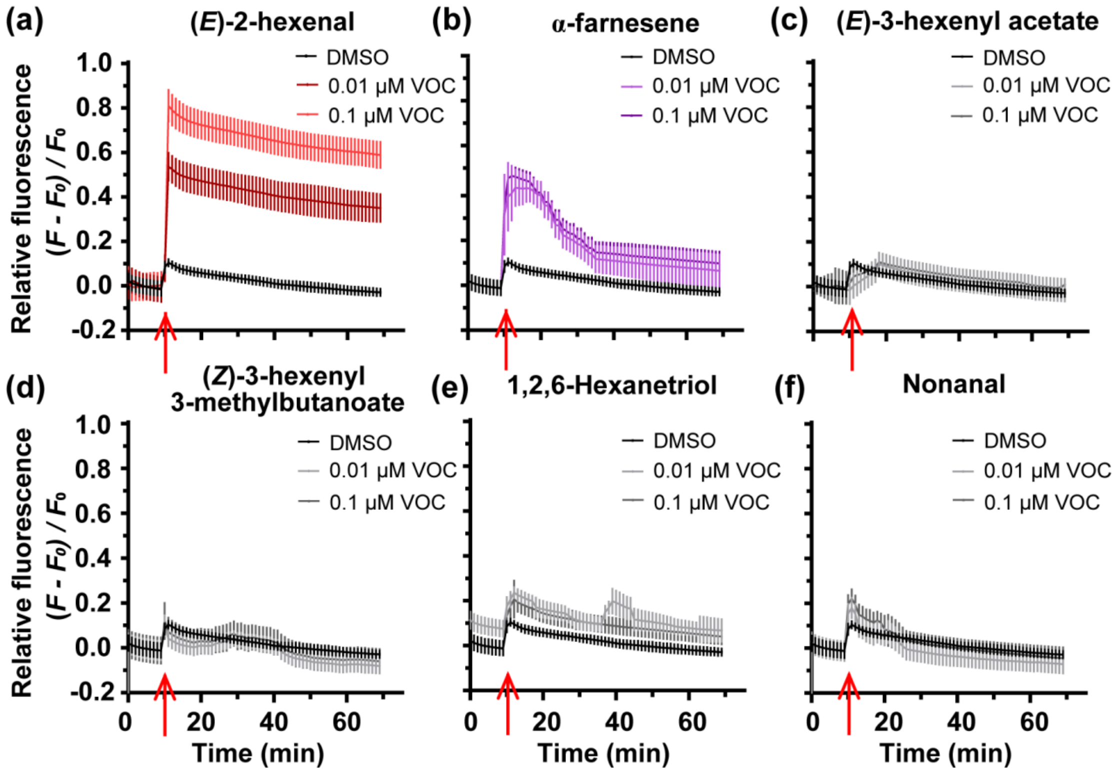

Fig. S6 High-throughput screening of $[Ca^{2+}]_{cyt}$-responsive volatiles induced by cold stress in tea leaf protoplasts

Time-course quantification of $[Ca^{2+}]_{cyt}$ dynamics in tea protoplasts treated with individual cold-induced volatiles: (*E*)-2-hexenal **(a)**, α-farnesene **(b)**, (*E*)-3-hexenyl acetate **(c)**, (*Z*)-3-hexenyl 3-methylbutanoate **(d)**, 1,2,6-hexanetriol **(e)**, nonanal **(f)**. Luminescence was recorded at 1-minute intervals using a multimode microplate reader (± SE, n = 4 replicates). The relative fluorescence intensity, calculated as $(F - F_0) / F_0$, was determined by normalizing the fluorescence intensity at each time point (F) to the baseline fluorescence intensity (F_0). The red arrow indicates the time point of reagent addition and reaction initiation.

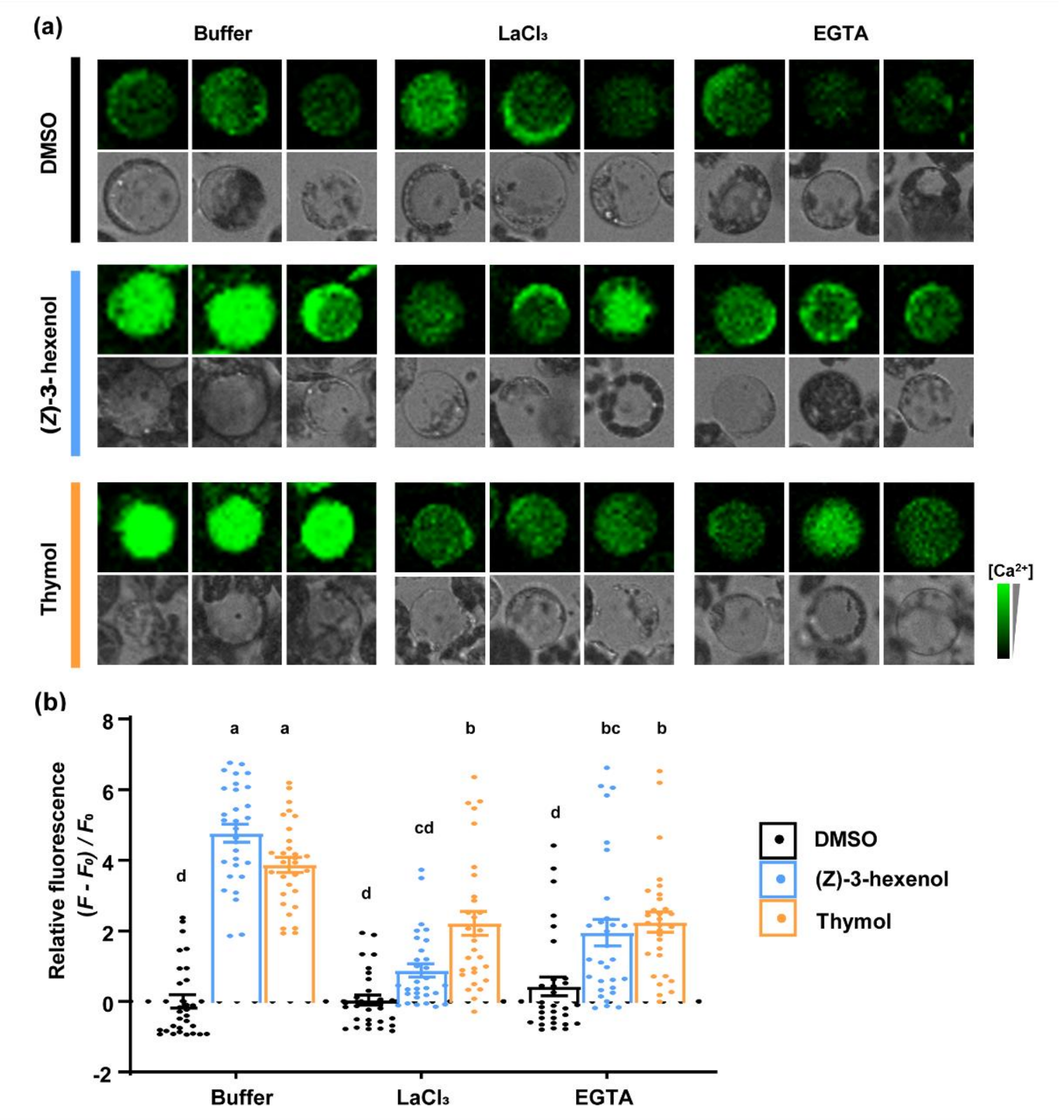

Fig. S7 Effects of calcium inhibitors on (*Z*)-3-hexenol- and thymol-induced $[Ca^{2+}]_{cyt}$ responses in tea leaf protoplasts

(a) Confocal imaging of $[Ca^{2+}]_{cyt}$ in tea protoplasts treated with 0.01 µM of (*Z*)-3-hexenol or thymol after pre-incubation with calcium inhibitors $LaCl_3$ or EGTA. Fluo-8 dye was used for detection, and DMSO served as the negative control. Fluorescence is shown using a pseudo-color scale. **(b)** Quantification of relative Ca^{2+} fluorescence intensity from (a) (± SE; n = 30 protoplast cells). The relative fluorescence intensity, calculated as $(F - F_0) / F_0$, was determined by normalizing the fluorescence intensity at each time point (F) to the baseline fluorescence intensity (F_0). Data points represent individual replicates. Different letters indicate significant differences at $P < 0.05$ (two-way ANOVA with Tukey's HSD post hoc test).

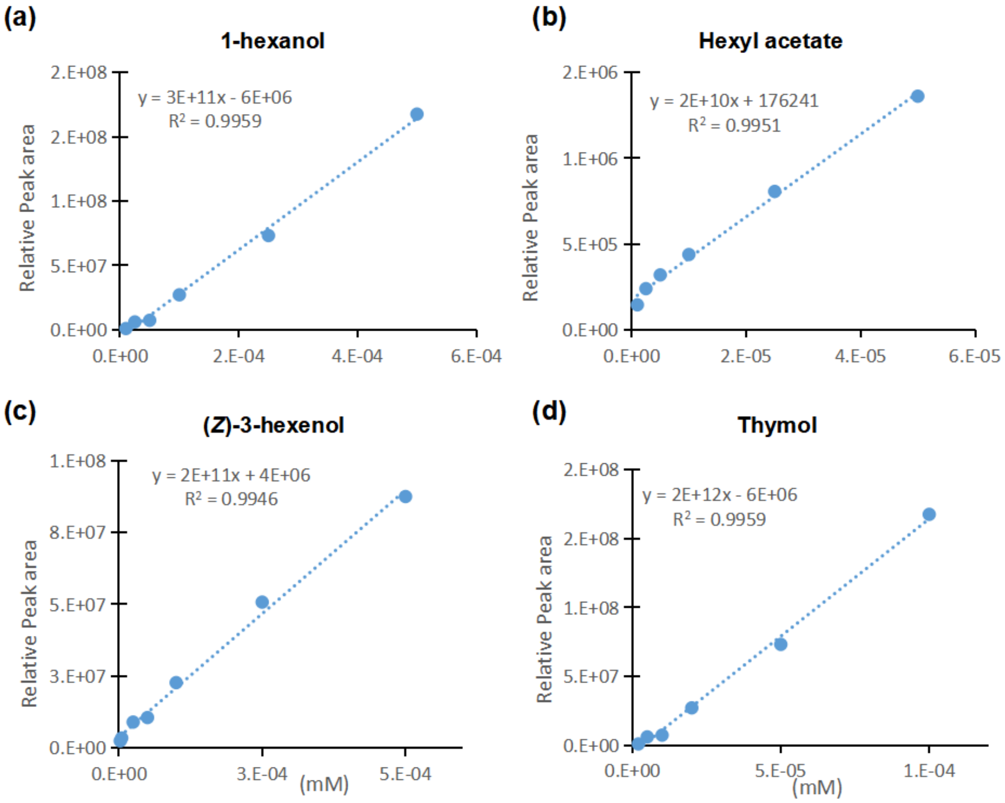

Fig. S8 Standard curves of 1-hexanol, hexyl acetate, (*Z*)-3-hexenol, and thymol

Individual volatile reagents at gradient concentrations were applied to cotton pieces, and the release rates of volatiles were quantified. A standard curve was generated based on the peak areas corresponding to the gradient concentrations.

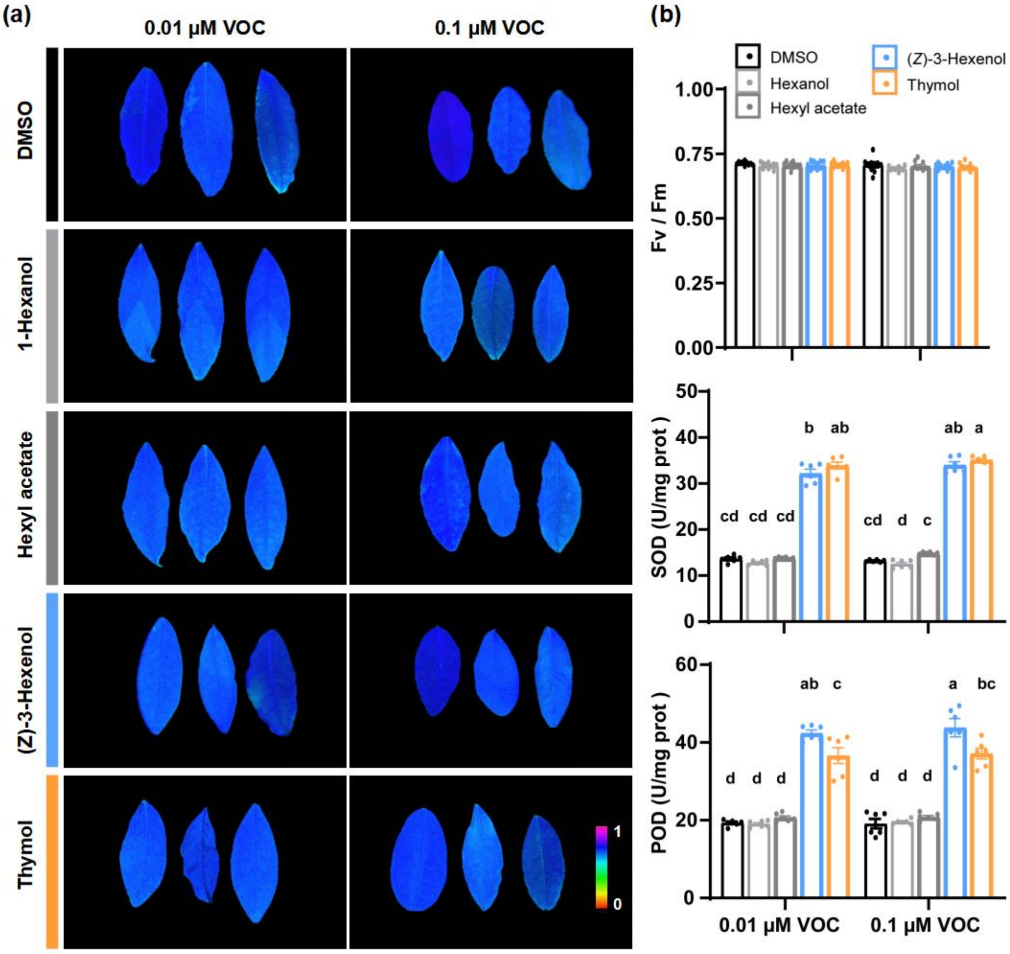

Fig. S9 [Ca^{2+}]$_{cyt}$-responsive volatiles (*Z*)-3-hexenol and thymol prime the antioxidant system in the absence of cold stress

(a) Chlorophyll fluorescence images of tea plants exposed to 1-hexanol, hexyl acetate, (*Z*)-3-hexenol, or thymol. DMSO was used as a negative control. Fv/Fm is presented using a pseudo-color scale ranging from 0 to 1. **(b-d)** Measurements of Fv/Fm (b), SOD activity (c) and POD activity (d) in tea plants treated with 1-hexanol, hexyl acetate, (*Z*)-3-hexenol, or thymol (± SE; n = 10 plants for Fv/Fm, n = 6 plants for SOD and POD activity). Data points represent individual replicates. Different letters indicate significant differences at $P < 0.05$ (two-way ANOVA with Tukey's HSD post hoc test).

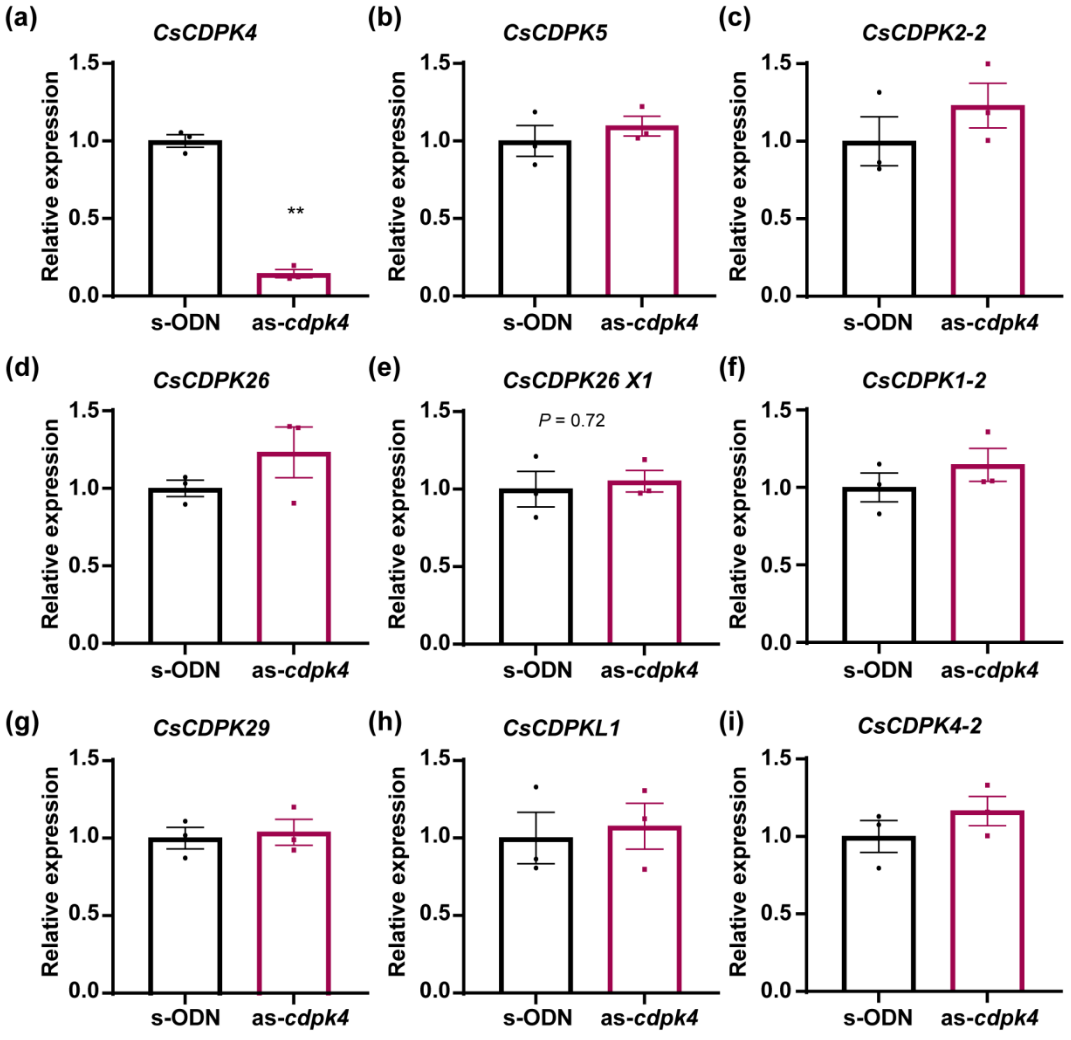

Fig. S10 Relative expression of *CsCDPK4* and its homologous genes in *CDPK4*-silenced tea plants

Relative expression levels of *CsCDPK4* **(a)** and its homologous genes *CsCDPK5* **(b)**, *CsCDPK2-2* **(c)**, *CsCDPK26* **(d)**, *CsCDPK26X1* **(e)**, *CsCDPK1*-2 **(f)**, *CsCDPK29* **(g)**, *CsCDPKL1* **(h)**, *CsCDPK4-2* **(i)** in control (s-ODN) and *CDPK4*-silenced (as-*cdpk4*) tea plants (± SE; *n* = 3 plants). Asterisks indicate significant differences compared to the control (***P* < 0.01, Student's *t* test).

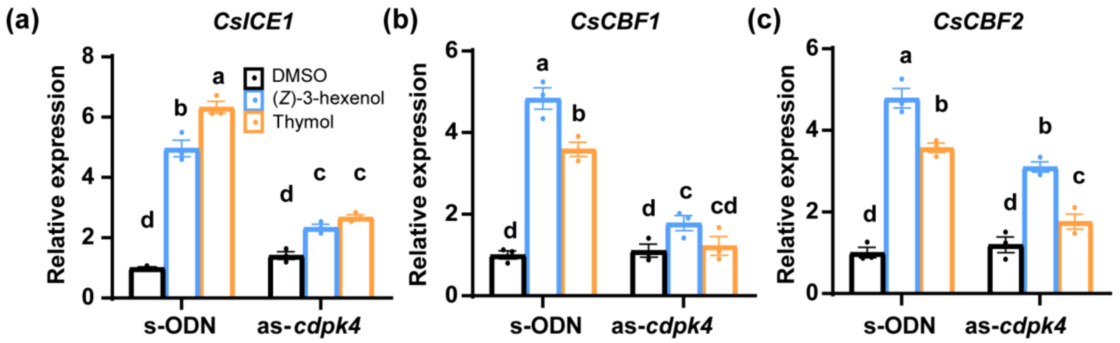

Fig. S11 CsCDPK4 is essential for (*Z*)-3-hexenol and thymol-induced cold stress tolerance in tea plants

Relative expression levels of cold-responsive genes *CsICE1* **(a)**, *CsCBF1* **(b)**, and *CsCBF2* **(c)** in control (s-ODN) and *CsCDPK4*-silenced (as-*cdpk4*) tea plants exposed to DMSO, 0.01 µM (*Z*)-3-hexenol, or thymol without cold stress treatment (± SE; n = 3 plants). Different letters indicate significant differences at $P < 0.05$ (two-way ANOVA with Tukey's HSD post hoc test).

Table S1 Volatile compounds emitted from tea plants in the recovery phase after cold stress

No.	Peak RT(min)	Volatile metabolite	Content （Peak Area）		Treatment/ control
			Treatment	control	
1	5.24	(*Z*)-2-pentenol	1780365	--	++
2	6.19	(*E*)-2-hexenal	301964420	708469	426.22
3	8.57	(*Z*)-3-hexenol	186254503	276339	674.01
4	9.14	(*Z*)-2-hexenol	3948318	108837	36.28
5	9.32	1-hexanol	5284620	--	++
6	10.93	Heptaldehyde	6218741	--	++
7	17.87	(*E*)-3-hexenyl Acetate	5838041	--	++
8	18.59	Hexyl acetate	950804	--	++
9	19.91	5-ethyl-2(5H)-furanone	5405341	--	++
10	23.19	1,2,6-hexanetriol	3553048	--	++
11	24.36	trans-linalool oxide	281412	--	++
12	25.78	Linalool	4881818	--	++
13	26	Nonanal	1100926	110995	9.92
14	32.37	Methyl salicylate	1453361	272842	5.33
15	33.8	Decanal	632113	186515	3.39
16	35.59	(*Z*)-3-hexenyl 3-methylbutanoate	133587	--	++
17	37.7	Geraniol	585856	--	++
18	42.22	Thymol	114173	--	++
19	49.6	α-farnesene	1561354	500031	3.12

The content of the main volatiles induced during the recovery phase after cold stress was detected using GC-MS. RT, retention time. Treatment/control represents the ratio of the volatile content of cold stressed plants to that of control. “++” indicates that the ratio is very significantly high due to the compound’s very low concentration in the control.

Table S2 Expression patterns of calcium-dependent protein kinases (*CDPKs*) in response to cold stress in tea plants

Table S3 Primers used for real time RT-PCR assays

GENE	ID	Primer pairs
CsICE1	CSS0026415	F:CGTCTTCGTCTTGTTCACCATC<br>R:CCAGATCGAAGCTGTGGTCTAA
CsCBF1	CSS0030784	F: AGAAATCGGATGGCTTGTGT<br>R: TTGTCGTCTCAGTCGCAGTT
CsCBF2	CSS0009762	F: CACAGCCTGCTCATCACT<br>R: ACCACTGCCACAATCTG
CsCDPK4	CSS0042249	F:TGAGAACACCCAGAAGCAACA<br>R:CCTTGGCCTAGCTTTTTGCC
CsCDPK17	CSS0042985	F:GAACTGTTGTTCCCATGGCG<br>R:ATGTTTTTAGGCGGGGTGGT
CsCDPK10	CSS0023979	F:AGATCATGCGAGAGGTTGACA<br>R:CACACACAGCTGTCCTGAGT
CsCDPK28	CSS0008031	F:GTGGGTCAAACAGCAACACC<br>R:GTTCTTCGCGGTGATTGTCG
CsCDPK30	CSS0003317	F:CGCCAGTATTCGAGGGAGAG<br>R:ACCACGACAGTTTGACCAGT
CsCDPK1	CSS0012731	F:ATCTCTCAACGGCACTCTCC<br>R:TTCGGGTTCGGGATTTGGAA
CsCDPK2	CSS0030469	F:CAACCCAGATGCAGGACAAA<br>R:TAATCTTCCCAGCTCCCACC
CsCDPK1L	CSS0006250	F:TGGGAAACGTCTGTGTTGGA<br>R:GCTAACGAAGGCACCTCACTA
CsCDPK5	CSS0023609	F:TTCAACAAACCCACCACCAG<br>R:GGGTCTTGTAGGGAAGGACC
CsCDPK2-2	CSS0009743	F:AACGACGGGCGCATAGATTA<br>R:CCCCTCTAAACCCAATGCTGA
CsCDPK26	CSS0004197	F:TGCCGTGGATCTTTTGGAGT<br>R:GTAGTAGTGGTGGTGGTGGTG
CsCDPK26X1	CSS0019429	F:CACATGCCGTGGATCTTTCG<br>R:CTGTTGGGTGAGTCATCGGA
CsCDPK1-2	CSS0040419	F:GGGGAATACTTGTGTTGGACCT<br>R:ACTTTCTCTATTAGTGGCGGAGG
CsCDPK29	CSS0016732	F:TCAAAGCGAAAGACCGGAGA<br>R:AGAGATGGGGCTGGTTTTGG
CsCDPKL1	CSS0013163	F:TGTTAGGGTGTAAACCTGCTTCT<br>R:ACAAGACGTTGATACATGCCTG
CsCDPK4-2	CSS0032244	F:GGTACAGGAAAAGGGAAAGCG<br>R:TTTCATGGCCTGTTGGGTTG
CsACTIN	CSS0050441	F: GATTCCGTTGCCCTGAAGTCCT<br>R: CCTTGCTCATACGGTCTGCGATA
